# Comprehensive Investigation on Controlling for CT Imaging Variabilities in Radiomics Studies

**DOI:** 10.1038/s41598-018-31509-z

**Published:** 2018-08-29

**Authors:** Rachel B. Ger, Shouhao Zhou, Pai-Chun Melinda Chi, Hannah J. Lee, Rick R. Layman, A. Kyle Jones, David L. Goff, Clifton D. Fuller, Rebecca M. Howell, Heng Li, R. Jason Stafford, Laurence E. Court, Dennis S. Mackin

**Affiliations:** 1 Department of Radiation Physics, The University of Texas MD Anderson Cancer Center, Houston, Texas USA; 2 MD Anderson Cancer Center UTHealth Science Center at Houston Graduate School of Biomedical Sciences, Houston, Texas USA; 3 Department of Biostatistics, The University of Texas MD Anderson Cancer Center, Houston, Texas USA; 4 Department of Imaging Physics, The University of Texas MD Anderson Cancer Center, Houston, Texas USA; 5 Medical & Radiation Physics, Inc, San Antonio, Texas USA; 6 Division of Radiation Oncology, The University of Texas MD Anderson Cancer Center, Houston, Texas USA

## Abstract

Radiomics has shown promise in improving models for predicting patient outcomes. However, to maximize the information gain of the radiomics features, especially in larger patient cohorts, the variability in radiomics features owing to differences between scanners and scanning protocols must be accounted for. To this aim, the imaging variability of radiomics feature values was evaluated on 100 computed tomography scanners at 35 clinics by imaging a radiomics phantom using a controlled protocol and the commonly used chest and head protocols of the local clinic. We used a linear mixed-effects model to determine the degree to which the manufacturer and individual scanners contribute to the overall variability. Using a controlled protocol reduced the overall variability by 57% and 52% compared to the local chest and head protocols respectively. The controlled protocol also reduced the relative contribution of the manufacturer to the total variability. For almost all variabilities (manufacturer, scanner, and residual with different preprocesssing), the controlled protocol scans had a significantly smaller variability than the local protocol scans did. For most radiomics features, the imaging variability was small relative to the inter-patient feature variability in non–small cell lung cancer and head and neck squamous cell carcinoma patient cohorts. From this study, we conclude that using controlled scans can reduce the variability in radiomics features, and our results demonstrate the importance of using controlled protocols in prospective radiomics studies.

## Introduction

Research interest in radiomics has been growing, as radiomics has shown promise in improving models for predicting patient outcomes. Radiomics involves evaluating images on a voxel-level basis on the assumption that there is more data to be extracted than can be observed by the human eye^1^. This process combined with conventional prognostic factors (e.g., age) has been able to improve survival models, demonstrated through the extensive studies in non–small cell lung cancer (NSCLC)^2–8^. Radiomics features for improving head and neck cancer models have recently been studied and have shown similar positive results of incorporating radiomics features in outcome models^9–14^.

Many of these radiomics studies are conducted at one facility. However, as the field of radiomics has grown, researchers have sought larger patient cohorts by combining data from multiple facilities. This means that patients are scanned on different computed tomography (CT) scanners using different protocols, which may affect radiomics features^15^. The impacts of differences in kernel, pixel size, and image thickness have been studied^16–21^. For parameters such as pixel size, it has been shown that resampling can reduce imaging differences^16,21^, while for parameters such as the reconstruction kernel, it has been shown that combining patient data that includes both sharp and smooth kernels can lead to large discrepancies^18^.

These uncertainty studies often involve only a few scanners at one facility, which provides valuable information about imaging variability, but these results may not be generalizable to a larger population of CT scanners at multiple facilities. Mackin *et al*. created a radiomics phantom to investigate the imaging variability among 17 scanners using the routine chest protocol on each^22^. They found that radiomics feature value differences due to the different scanners were similar to the inter-patient radiomics feature variability among NSCLC patients and thus recommended that these imaging differences be considered in future studies.

In this study, we aimed to obtain a large sample of CT scanners for an in-depth analysis of imaging variability to determine how retrospective radiomics studies should select patients and how prospective radiomics studies should design CT protocols. The large sample would allow for the conclusions to be applied generally to all CT scanners. Local protocols were used, as many studies use retrospective data and it is of interest whether protocol differences will cause large radiomics feature value differences, thus causing patient stratification to be dominated by scan protocol and not true patient radiomics feature values. Also, a controlled scan was used to see whether imaging differences could be minimized using a harmonized protocol across different vendors.

## Methods

### Materials

**Figure 1:**
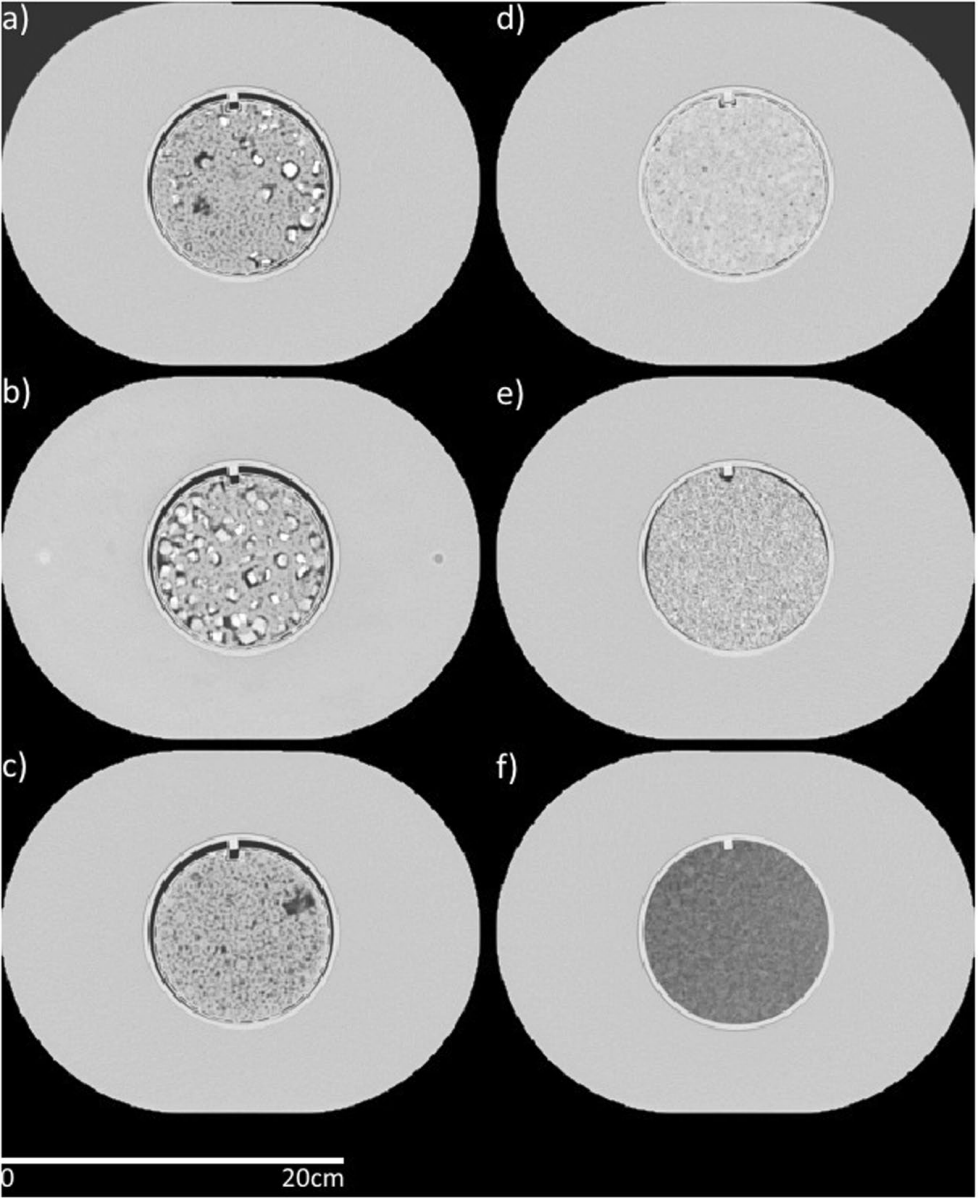
Axial views from a computed tomography scan of the radiomics phantom used. The cartridges are (**a**) 50% acrylonitrile butadiene styrene (ABS), 25% acrylic beads, and 25% polyvinyl chloride (PVC) pieces (percentages are by weight), (**b**) 50% ABS and 50% PVC pieces, (**c**) 50% ABS and 50% acrylic beads, (**d**) hemp seeds in polyurethane, (**e**) shredded rubber, and (**f**) dense cork. The high-density polystyrene buildup is seen outside the cartridges with dimensions of 28 cm × 21 cm × 22 cm. The cartridges had a diameter of 10.8 cm. Window width: 1600, window level: −300.

We used an updated version of the Credence Cartridge Radiomics phantom originally described by Mackin *et al*.^22^ in 2015. This version of the phantom, shown in Fig. 1, is comprised of six round cartridges encased in high-density polystyrene buildup. The six cartridges were held within the buildup in an acrylic case with a notch designed to keep the cartridges in the same position. This case can be seen in Fig. 1 as the bright line around each cartridge before the buildup. The size of the buildup, 28 cm × 21 cm × 22 cm, is based on the mean physical dimensions of a European woman’s chest^23^. The six cartridges are each comprised of different materials: 50% acrylonitrile butadiene styrene (ABS), 25% acrylic beads, and 25% polyvinyl chloride (PVC) pieces (percentages are by weight); 50% ABS and 50% PVC pieces; 50% ABS and 50% acrylic beads; hemp seeds encased in polyurethane; shredded rubber; and dense cork. These materials were chosen to produce a range of radiomics feature values similar to those of NSCLC tumors for the original materials^22^, the new materials followed the same analysis as the original materials. Additional details on the differences between this phantom and the original phantom are described in the Discussion.

### CT Scans

A controlled CT scan was acquired using the following parameters for each scanner: tube voltage, 120 kV(p); tube current, 200 mA∙s; helical scan type; spiral pitch factor, 1.0; 50-cm display field of view; and image thickness, 3 mm (except for GE scanners, which used an image thickness of 2.5 mm). The acquisition parameters were designed to give about 13 mGy CTDI_vol_ (average 16 mGy, standard deviation 4 mGy) in order to produce the same noise characteristics. A recent study by Mackin *et al*.^24^ showed that features were not affected by noise levels in the image, thus variations in CTDI_vol_ should not impact the radiomics features. The convolution kernel was standard for GE; C for Philips; B31f, B31s for Siemens; and FC08 for Toshiba. These kernels were chosen to minimize the difference in radiomics feature values across vendors as described in Mackin *et al*.’s abstract^25^. Also, the local chest protocol and local head and neck protocol were used to acquire scans of the phantom. For the local protocols, no parameters were changed in order to estimate the variability in protocols across institutions and scanners. The parameters for each of the local protocol scans is supplied in the Supplemental Material.

### Patient Scans

A phantom alone cannot provide insight into the impact of feature variability within a patient study. Thus, we have included patient cohorts to determine the size of the imaging variability with respect to inter-patient variability, providing an estimate on the impact of the imaging variability for each feature.

For this study, we retrospectively reviewed the images and medical records of 20 patients with NSCLC and 30 patients with head and neck squamous cell carcinoma (HNSCC) with a waiver of informed consent from the Institutional Review Board at the University of Texas MD Anderson Cancer Center. These two cohorts of patients were used to compare the imaging variability to inter-patient variability. Radiomics features have been shown to improve the patient outcome models for both of these patient types^2,5,10,12,26^.

The NSCLC cohort had 10 men and 10 women, mean age of 67 years (range, 52–78 years), mean weight of 72.9 kg (range, 41.0–97.6 kg), and mean height of 170 cm (range, 154–182 cm). The CT scans were acquired on a GE Discovery CT scanner (GE Healthcare, Little Chalfont, UK) at 120 kVp, 300 mA, 0.5 s rotation time, 2.5-mm image thickness, 1.35 pitch, and 0.976 mm × 0.976 mm pixel size.

The HNSCC cohort had 25 men and 5 women, mean age of 64 years (range, 50–87 years), mean weight of 80.5 kg (range, 43.9–114.9 kg), and mean height of 175 cm (range, 149–193 cm). The CT scans were acquired using a GE LightSpeed CT scanner at 120 kVp, 220 mA, 1.0 s rotation time, 1.25-mm image thickness, 1.375 pitch, and 0.488 mm × 0.488 mm pixel size. For both patient cohorts, the tumors were contoured by a radiation oncologist.

### Radiomics Feature Extraction

The phantom was semi-automatically contoured using an in-house MATLAB (version 2016b, MathWorks, Natick, MA, USA) script. A cylindrical region of interest (ROI) was created for each cartridge. Each ROI was 8.2 cm in diameter. The ROIs for the cartridge with 50% ABS and 50% acrylic beads and the cartridge with hemp seeds in polyurethane each had a height of 1.9 cm. All other ROIs each had a height of 2 cm. Mackin *et al*. showed that the size of the ROI did not impact conclusions of a phantom study^24^, therefore we maximized the acceptable region within each cartridge. The ROIs were automatically placed into IBEX, an open-source radiomics tool^27,28^, and then viewed to determine acceptability. Generated contours were scrutinized and edited as needed.

Forty-nine features were calculated using IBEX: 22 gray level co-occurrence matrix features^29^, 11 gray level run length matrix features^30,31^, 11 intensity histogram features, and five neighborhood gray tone difference matrix features^32^ (Table 1). Four different preprocessing techniques were used for each feature: (1) thresholding; (2) thresholding and 8-bit depth resampling; (3) thresholding and a Butterworth smoothing filter (order of 2, cut-off of 125); and (4) thresholding, 8-bit depth resampling, and Butterworth smoothing^33^. The thresholds for the NSCLC patient cohort were a lower threshold of −100 HU and a higher threshold of 200 HU. A lower threshold of −100 HU was used for the HNSCC patient cohort with no upper threshold. No thresholding was applied to the phantom images. The settings for each feature were the same as those listed by Fave *et al*. in the Supplemental Material^3^. For the local scans, the pixel size was resampled to 1 mm × 1 mm using trilinear interpolation as suggested by the results from Mackin *et al*.^21^. For features that have been previously found to correlate with volume, the updated formulae were used as described by Fave *et al*.^33^.

**Table 1:** Radiomics Features Analyzed.

Gray Level Co-occurrence Matrix	Gray Level Run Length Matrix	Intensity Histogram	Neighborhood Gray Tone Difference Matrix
Auto Correlation	Gray Level Nonuniformity	Energy	Busyness
Cluster Prominence*	High Gray Level Run Emphasis	Entropy	Coarseness
Cluster Shade*	Long Run Emphasis	Kurtosis	Complexity
Cluster Tendency	Long Run High Gray Level Emphasis	Maximum	Contrast
Contrast	Long Run Low Gray Level Emphasis	Mean	Texture Strength
Correlation	Low Gray Level Run Emphasis	Median	
Difference Entropy	Run Length Nonuniformity	Minimum	
Dissimilarity	Run Percentage	Skewness*	
Energy	Short Run Emphasis	Standard Deviation	
Entropy	Short Run High Gray Level Emphasis	Uniformity	
Homogeneity	Short Run Low Gray Level Emphasis	Variance	
Homogeneity 2			
Information Measure Correlation 1			
Information Measure Correlation 2			
Inverse Difference Moment Norm			
Inverse Difference Norm			
Inverse Variance			
Max Probability			
Sum Average			
Sum Entropy			
Sum Variance			
Variance			

* indicates features that were subsequently not used due to sensitivity of region of interest placement within the phantom material.

### Statistical Methods

#### Feature Stability

The features were tested for reproducibility by moving the ROIs on one controlled scan of the phantom. The ROIs were shifted 10 times within the acceptable region of the cartridges. The coefficient of variation was calculated for each feature. Features for which more than 50% of instances (with four preprocessing types and six cartridges, there were 24 total instances for each feature) had a coefficient of variation above 10% were removed from further analysis. It was important to remove these features as features that are very sensitive to the positioning of the ROI may not properly represent the imaging variation and may only represent placement of the ROI on the different scans.

#### Resampling the z Dimension

For the local protocol scans, the image thickness was not consistent. The impact of the image thickness on feature value was evaluated by computing the Pearson correlation for each ROI-feature combination. Additionally, the impact of resampling the image thickness was investigated by resampling the z dimension from 1 mm to 7 mm in 1 mm increments. Features were acquired using all z dimension resampling values and without resampling the z dimension. The intra-class correlation coefficient (ICC) was computed for each feature using the eight resampling options to determine if resampling changed the feature values and thus reduced the correlation of feature values with image thickness. The ICC (2, 1) (two-way random effects, absolute agreement, single rater/measurement) and ICC (3, 1) (two-way random effects, consistency, single rater/measurement) as described by Shrout and Fleiss^34^ were computed in R (version 3.4.3) using the psych package (version 1.7.8)^35^. For these tests, features were calculated with thresholding preprocessing on the local chest protocol scans. The other preprocessing techniques and the head protocol scans were not used as this step was simply to determine the relationship between image thickness and feature values, and the additional preprocessing and protocol scans produced redundant data.

#### Imaging Variability

Our goal was to determine how the manufacturer and scanner uncertainties contribute to the overall variability in the feature values. To determine these uncertainties, we first built a linear mixed-effects model, which estimates the contribution of the manufacturer, the additional scanner-wise variability within a given manufacturer, the cartridge material, and the residual to the variability in the measurements. The standard deviations of the distributions are used to provide estimates of the variability contributed from the manufacturer, scanner, cartridge material, and residual. The term scanner is used here to indicate an individual scanner (e.g., multiple of the same type of scanner from the same manufacturer are each considered distinct). There are many factors that could affect the images from a particular scanner, including the quality assurance (QA) technique/periodicity, scanner maintenance, and scanner design. Thus, radiomics features calculated from images taken using CT scanners of the same manufacturer/model may be different. The term residual typically implies a small contribution. However, for this study the term is simply used to represent anything that is not included within the formula (i.e., anything that is unknown).

A linear mixed-effects model was created for each scan type (control, local chest, and local head and neck protocol):

1 $${f}_{m,i}=\mu +{\alpha }_{m}+{\beta }_{i}+g(t)+{\varepsilon }_{m,i},$$

where *f* is the feature, µ is the mean, *m* is the cartridge material, *i* is the scanner, α is the material-wise contribution, β is the scanner-wise contribution, *g*(*t*) is the fixed effect of the impact of image thickness on feature value, and ε is the residual. β_i_ is normally distributed with a mean of γ_v,i_ and a variance of ${\sigma }_{\beta ,m}^{2}\,({\sigma }_{\beta ,m}^{2}={\sigma }_{\beta }^{2}\times {\hat{f}}_{m}^{2})$. γ_v,i_ is the vendor-wise contribution which is normally distributed with a mean of 0 and a variance of ${\sigma }_{\gamma ,m}^{2}({\sigma }_{\gamma ,m}^{2}={\sigma }_{\gamma }^{2}\times {\hat{f}}_{m}^{2})$. $\hat{{f}_{m}}$ is the mean feature value for the cartridge material. ε_m,i_ is normally distributed with a mean of 0 and variance of ${\sigma }_{\varepsilon ,m}^{2}({\sigma }_{\varepsilon ,m}^{2}={\sigma }_{\varepsilon }^{2}\times {\hat{f}}_{m}^{2})$ The model computes a significance test before producing the results. If the standard deviation due to one component is much smaller than the others, it is set to 0 and combined into the residual. The linear mixed-effects models were computed in R (version 3.4.3) using the lme4 package (version 1.1–17).

Imaging variability was measured using the uncertainties from the linear mixed-effects models. Currently, most studies do not apply corrections for the manufacturer and scanner. The total imaging variability was calculated to estimate the impact of continuing to not apply corrections. It was calculated as follows:

2 $$I{V}_{total}=\frac{{\sigma }_{t,m}/\widehat{{f}_{m}}}{{\sigma }_{p}/{\mu }_{p}},$$

where σ_p_ is the standard deviation of the feature value for patients, µ_p_ is the mean feature value for patients, and σ_t,m_ is the total standard deviation from the model, given by

3 $${\sigma }_{t,m}=\sqrt{{\sigma }_{\beta ,m}^{2}+{\sigma }_{\gamma ,m}^{2}+{\sigma }_{\varepsilon ,m}^{2}}.$$

This metric (equation 2) includes a comparison to the patients to gauge the impact of the imaging variability in a patient setting.

The residual imaging variability was calculated to estimate the imaging variability that would exist in cohorts that include CT images from different scanners even if corrections could be applied based on the manufacturer and individual scanner, as follows:

4 $$I{V}_{residual}=\frac{{\sigma }_{\varepsilon ,m}/\widehat{{f}_{m}}}{{\sigma }_{p}/{\mu }_{p}}.$$

We repeated this modeling process for the three scan types (control, local chest, and local head and neck protocols) and compared the results. To determine if the controlled scan significantly reduced the variability, we performed one-sided pairwise t-tests comparing σ_β_, σγ, and σ_ε_ between the controlled protocol and both local protocols.

#### Quality Assurance Using a Radiomics Phantom

The feasibility of creating a credentialing phantom for radiomics studies, similar to the credentialing of institutions for National Institutes of Health radiation therapy studies, was investigated. Ideally, the credentialing phantom would be small for ease of transport and use. Therefore, the ability of each cartridge was tested for its use in QA checks to determine which CT scanners do not fall within the credentialed standard population of scanners. The spread of feature values from different scanners should be small relative to the inter-patient spread, therefore, the patient standard deviations were used to determine if scanners fell close enough to the population scanner value or not. The controlled scans were used for this analysis. For each feature, the patient standard deviation was scaled to account for differences in means between the patient and phantom populations.

5 $${\sigma }_{scaled}=\frac{{\sigma }_{p}}{{\mu }_{p}}\times \hat{f}$$

For each scanner, the number of features that fell outside 1/3 of the scaled patient standard deviation from the mean feature value was tallied. The idea of the bounds was to determine if criteria could be established such that a certain number of features would fall within the bounds in order for the given scanner to pass the QA test. Therefore, the bounds were set as follows:

6 $$Lower\,bound=\hat{f}-\frac{1}{3}{\sigma }_{scaled}$$

7 $$Upper\,bound=\hat{f}+\frac{1}{3}{\sigma }_{scaled}$$

## Results

### Scanners

The phantom was scanned on 100 scanners: 51 GE scanners (GE Healthcare), 20 Philips scanners (Philips Healthcare, Eindhoven, the Netherlands), 17 Siemens scanners (Siemens Healthineers, Erlangen, Germany), 11 Toshiba scanners (Canon Medical Systems USA, Tustin, CA, USA), and one Philips and Neusoft Medical System scanner (Shenyang, China). Ninety-four scanners had a controlled protocol scan that could be used: 48 GE, 18 Philips, 17 Siemens, and 11 Toshiba scanners. However, the kernel used for the Toshiba scans switched from FC18 (six scanners) to FC08 (five scanners) halfway through owing to a study that found the FC08 kernel to match the GE standard kernel best^25^. To determine whether both Toshiba kernels could be used in the analysis, k-means clustering was performed. The scanners did not cluster by kernel type. While the best match should always be used to minimize discrepancies, in this study the kernel differences among the Toshiba scanners was not a driving force in the variability and therefore, kernel did not matter for Toshiba and all Toshiba scans were included in the analysis. Ninety-three scanners had a local chest protocol scan that could be used: 47 GE, 19 Philips, 17 Siemens, and 10 Toshiba scanners. Eighty-eight scanners had a local head protocol scan that could be used: 46 GE, 18 Philips, 14 Siemens, and 10 Toshiba scanners. The various reasons that scans could not be used were as follows: the field of view did not encompass all the cartridges, the scan extent did not cover the length of the phantom, and the scan was acquired with variable image thickness. Head and neck protocols could be acquired only on CT scanners used for radiation therapy purposes; on diagnostic scanners, a head scan, typically brain, was acquired (both head and neck and head protocols are referred to as “head protocols” hereafter).

We were able to ascertain that at least 96% of scanners followed AAPM or ACR recommendations for QA. Additionally, at least 49% of scanners were ACR accredited, 20% of scanners were in the radiation therapy department of scanners at ACR accredited facilities, and 6% of scanners were currently undergoing ACR accreditation.

**Figure 2:**
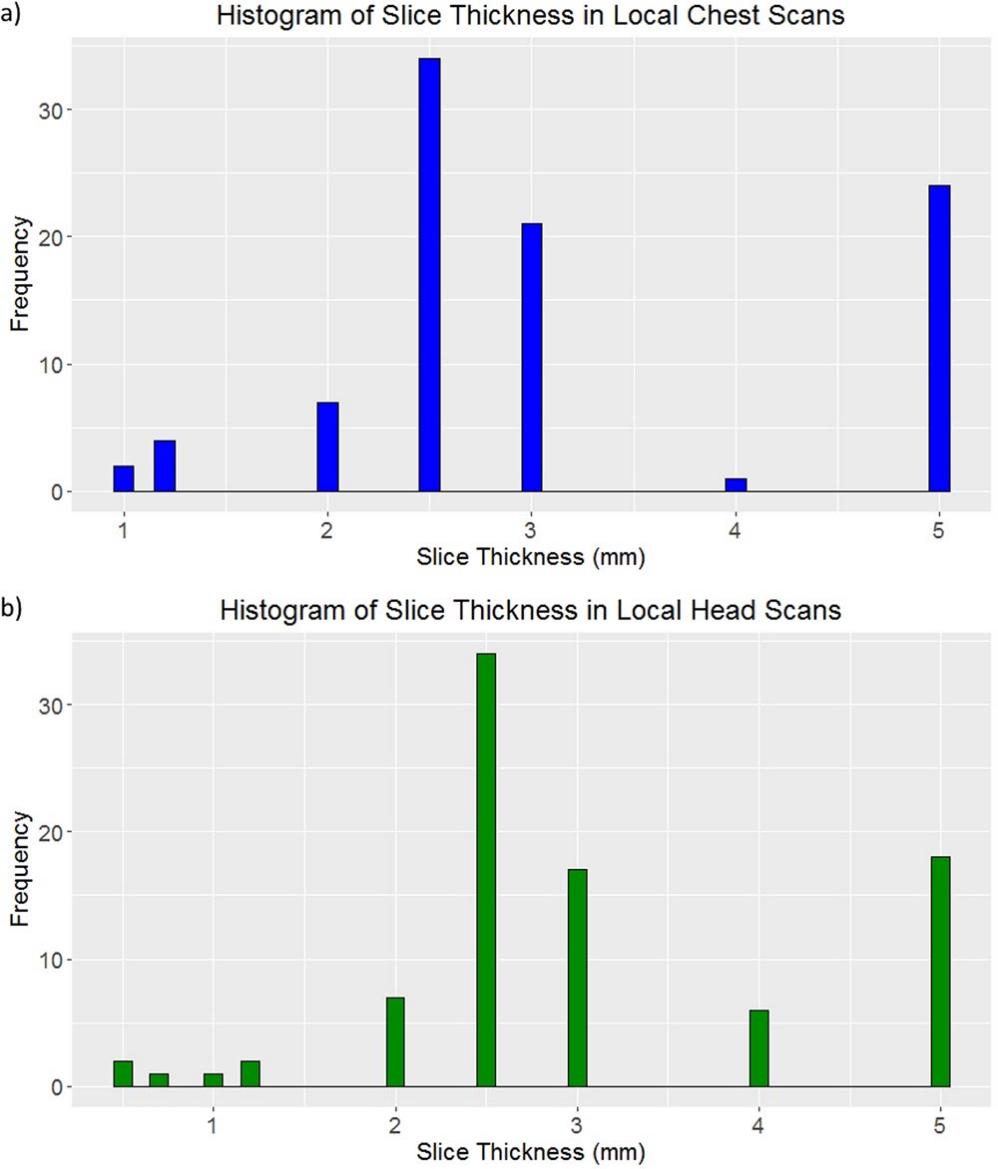
Histograms of image thicknesses across the scans taken using (**a**) the local chest protocol and (**b**) the local head protocol.

The local chest protocol scans had image thicknesses ranging from 1 to 5 mm. The local head protocol scans had image thicknesses ranging from 0.5 to 5 mm. Histograms of the distributions are shown in Fig. 2.

### Feature Stability

Three features had a coefficient of variation greater than 10% in more than 50% of instances (with 24 total instances for each feature): the features of cluster prominence, cluster shade, and skewness. These features were not included in subsequent analysis. The coefficient of variation exceeded 10% for auto correlation and sum variance in 42% of instances and for long run low gray level emphasis, low gray level run emphasis, short run low gray level emphasis, and the minimum in 46% of instances. All other features had a coefficient of variation greater than 10% in less than 25% of instances; the majority of features had a coefficient of variation greater than 10% in 0% of instances.

### Resampling the *z* Dimension

**Figure 3:**
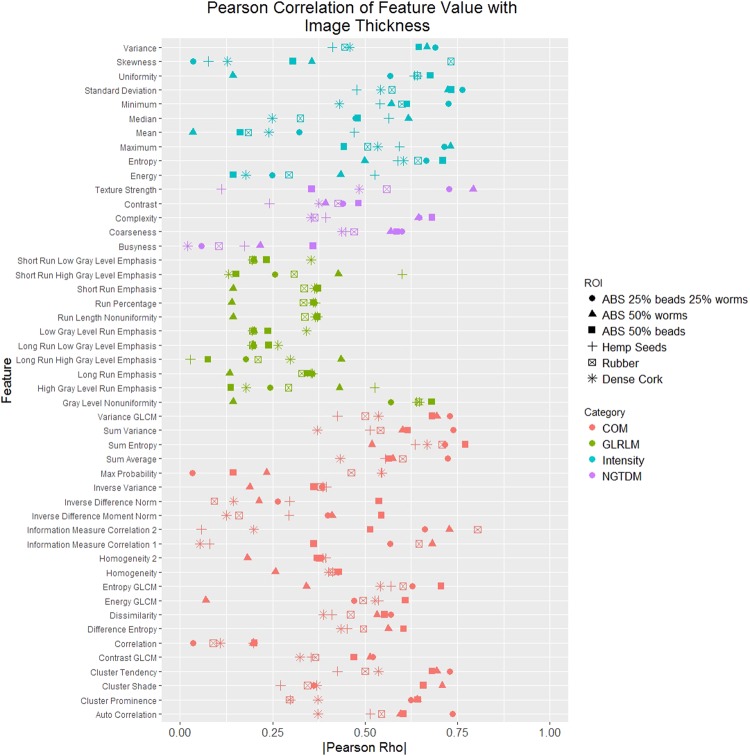
Absolute value of the Pearson correlation rho for the correlation between feature value and image thickness for each region of interest (ROI). Each ROI is a different shape. Each category of feature is a different color. The correlation varies between and within features depending on the ROI. COM: gray level co-occurrence matrix, GLCM: gray level co-occurrence (used when there are features with the same name in different categories to differentiate them), GLRLM: gray level run length matrix, NGTDM: neighborhood gray tone difference matrix, beads: acrylic beads, worms: polyvinyl chloride pieces.

Figure 3 shows the absolute value of the Pearson correlation coefficient of each ROI for the correlation of each feature with the image thickness. The mean absolute value of the Pearson correlation coefficient was 0.42. The correlation values had similar ranges for all the feature categories except for the gray level run length matrix category, which had lower correlation values. The mean absolute value of the Pearson correlation coefficient increased to 0.46 when gray level run length matrix features were not included. A second version of Fig. 3 without the ABS cartridges is repoduced in the Supplemental Material. For this analysis the mean absolute value of the Pearson correlation coefficient was 0.39. Without the gray level run length matrix features, the mean absolute value of the Pearson correlation coefficient was 0.41.

To determine the level of reliability based on the ICC values, the guidelines from Koo and Li were followed^36^. ICC values less than 0.5 signify poor reliability, those between 0.5 and 0.75 signify moderate reliability, those between 0.75 and 0.9 signify good reliability, and those greater than 0.9 signify excellent reliability. When comparing feature values across different resampling techniques using ICC (2, 1) (two-way random effects, absolute agreement, single rater/measurement), we found that 35 features had excellent reliability, seven features had good reliability (entropy, max probability, low gray level run emphasis, short run low gray level run emphasis, busyness, complexity, and contrast), and four features had moderate reliability (information measure correlation 1, information measure correlation 2, long run low gray level emphasis, and texture strength). When ICC (3, 1) (two-way random effects, consistency, single rater/measurement) was used, we found that 39 features had excellent reliability, five features had good reliability (information measure correlation 2, max probability, low gray level run emphasis, short run low gray level run emphasis, and texture strength), one feature had moderate reliability (long run low gray level emphasis). Thus, feature values did not change with resampling; therefore, for the linear mixed-effects analysis, no resampling in the *z* dimension was done for the local chest and local head protocols. Additionally, these results paired with the Pearson correlation results implied that there was a relationship with image thickness that needed to be included in the modeling.

### Imaging Variability

**Figure 4:**
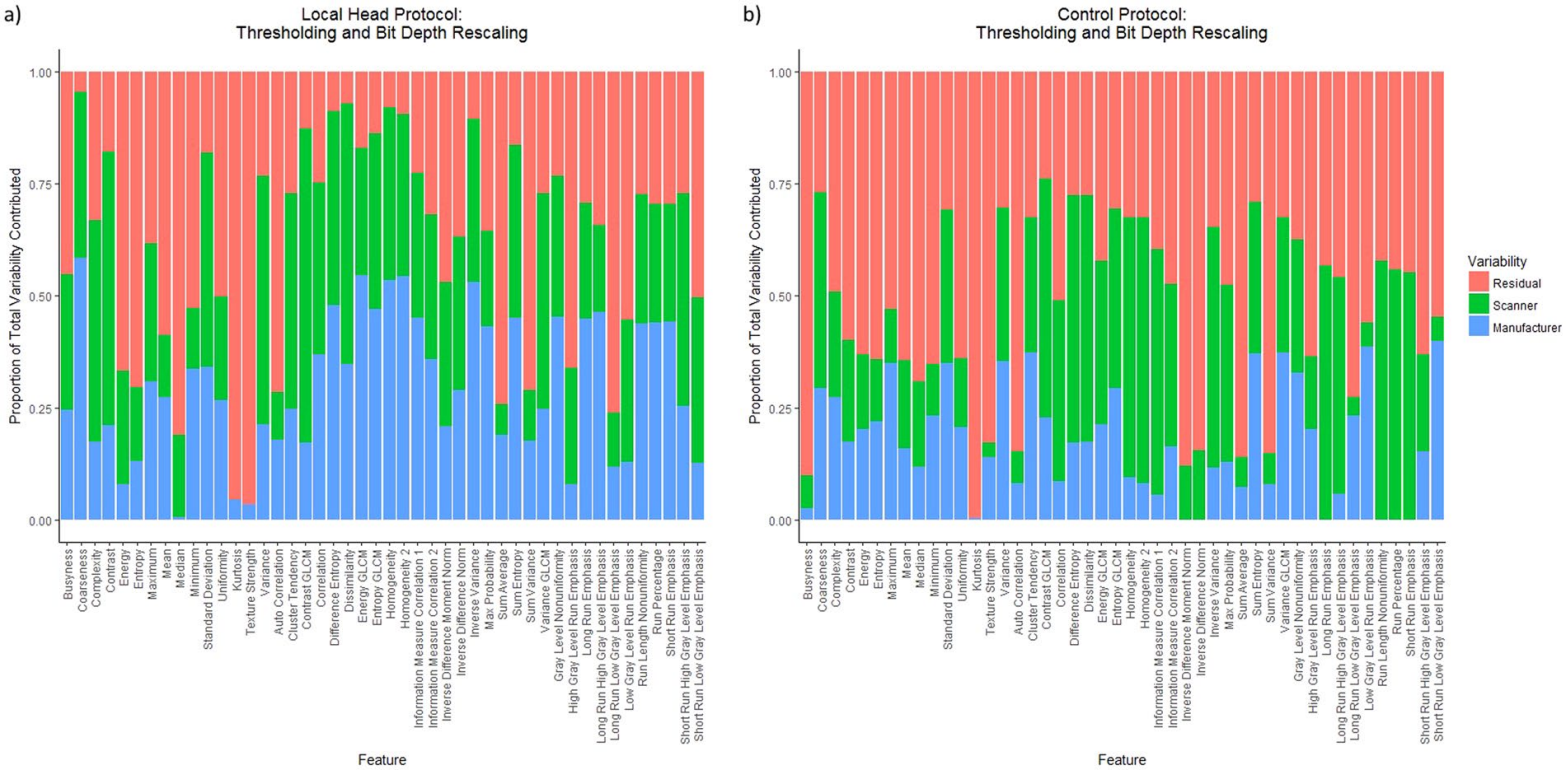
Bar plots of the relative contributions of the scanner-wise variability (green), manufacturer-wise variability (blue), and residual variability (red) for each feature using thresholding and bit depth rescaling calculated on (**a**) the local head protocol and (**b**) the controlled protocol. The contribution of the manufacturer was much larger for many features in the local head protocol than in the controlled protocol. The total variability for the controlled protocol compared with that of the head protocol was 0.48.

The variability due to the material was 0 in every model. The relative proportions of σ_β_ (scanner-wise variability), σ_γ_ (manufacturer-wise variability), and σ_ε_ (residual variability) were calculated for each feature. Plots of the proportion of each of these variabilities using thresholding and bit depth rescaling are shown in Fig. 4 for the controlled protocol and local head protocol. All other plots (other preprocessing and chest protocol) are in Supplemental Figs 2–11. Figure 4 shows that the contribution from σ_γ_ is reduced when the controlled protocol is used. The mean total variability for the controlled protocol was 0.43 compared with that of the local chest protocol and was 0.48 compared with that of the local head protocol. The average proportion of total variability was 0.29, 0.27, and 0.43 for the manufacturer, scanner, and residual respectively based on the head protocol scans. The average proportion of total variability was 0.30, 0.27, and 0.44 for the manufacturer, scanner, and residual respectively based on the chest protocol scans. The average proportion of total variability was 0.20, 0.25, and 0.55 for the manufacturer, scanner, and residual respectively based on the controlled protocol scans. The details of this are shown in Fig. 4.

The residual contribution was not always small; it was often the largest component. This is particularly evident for the controlled protocol where the residual should have a large relative contribution since factors that were contributing to the variability have been accounted for in the design of the protocol. The manufacturer contribution was not always larger than the scanner contribution to the total variability, as can be seen in Fig. 4, thus demonstrating that the variability among scanners of the same manufacturer can vary more than different manufacturers.

If it was possible to correct for the manufacturer and individual scanner, then, when using a controlled protocol, only the residual variability would remain. In that situation, the mean controlled residual variability would be 0.36 compared with the chest protocol total variability and 0.40 compared with the head protocol total variability. This is the theoretical best possible improvement that can be achieved until we have an in-depth understanding of the components hidden in the residual. In comparison to the controlled protocol, this is an additional 7–8% reduction in variability ($100\times (mean(\frac{total\,variability\,controlled\,protocol}{total\,variability\,local\,protocol})\,\,\,-mean(\frac{residual\,variability\,controlled\,protocol}{total\,variability\,local\,protocol})).$

The linear mixed-effects models produced a spectrum of variabilities, from high to low. For ease of summary, a cutoff has been established. Spreadsheets with the data are in the Supplemental Material to allow for different cutoffs to be used in future studies. For IV_total_ and IV_residual_ (equations 2 and 4), a cutoff of 1/3 was used to create a binary of significance (i.e. significant or not). This was done for each feature to indicate that the imaging variation was negligible relative to inter-patient variability or imaging variability was significant relative to inter-patient variability. The total numbers of features in each category that had IV_total_ or IV_residual_ values greater than 1/3 are displayed in Table 2.

**Table 2:** Number of features for each protocol and preprocessing type that have imaging variability compared to inter-patient variability from linear mixed-effects models above the cutoff.

Protocol	Feature Group	Thresholding				Thresholding and Smoothing				Thresholding and Bit Depth Rescaling				Thresholding, Smoothing, and Bit Depth Rescaling			
		Total Variability		Residual Variability		Total Variability		Residual Variability		Total Variability		Residual Variability		Total Variability		Residual Variability	
		NSCLC Patients	HNSCC Patients	NSCLC Patients	HNSCC Patients	NSCLC Patients	HNSCC Patients	NSCLC Patients	HNSCC Patients	NSCLC Patients	HNSCC Patients	NSCLC Patients	HNSCC Patients	NSCLC Patients	HNSCC Patients	NSCLC Patients	HNSCC Patients
Controlled Protocol	GLCM (N = 20)	1	1	1	1	0	2	0	2	0	0	0	0	1	3	1	3
	GLRLM (N = 11)	3	3	3	3	2	2	2	2	3	3	3	3	3	3	3	3
	Intensity (N = 10)	1	1	1	1	1	1	1	1	1	1	1	1	1	1	1	1
	NGTDM (N = 5)	0	0	0	0	0	0	0	0	0	0	0	0	0	0	0	0
Local Chest Protocol	GLCM (N = 20)	3	4	2	3	3	3	2	3	2	4	2	2	2	2	2	2
	GLRLM (N = 11)	3	3	3	3	3	3	3	3	3	3	3	3	2	2	2	2
	Intensity (N = 10)	1	1	1	1	1	1	1	1	1	1	1	1	1	1	1	1
	NGTDM (N = 5)	0	0	0	0	0	0	0	0	0	0	0	0	0	0	0	0
Local Head Protocol	GLCM (N = 20)	2	4	1	3	2	3	2	3	1	4	0	2	1	2	1	2
	GLRLM (N = 11)	3	3	3	3	3	3	3	3	3	3	3	3	1	1	1	1
	Intensity (N = 10)	1	1	1	1	1	1	1	1	1	1	1	1	1	1	1	1
	NGTDM (N = 5)	0	0	0	0	0	0	0	0	0	0	0	0	0	0	0	0

GLCM: gray level co-occurrence matrix, GLRLM: gray level run length matrix, NGTDM: neighborhood gray tone difference matrix, NSCLC: non–small cell lung cancer, HNSCC: head and neck squamous cell carcinoma. Total variability: $I{V}_{total}=\frac{{\sigma }_{t,m}/\widehat{{f}_{m}}}{{\sigma }_{p}/{\mu }_{p}}$, residual variability: $I{V}_{residual}=\frac{{\sigma }_{\varepsilon ,m}/\widehat{{f}_{m}}}{{\sigma }_{p}/{\mu }_{p}}$, with a cutoff of 1/3.

Two gray level run length matrix features and one intensity feature were always above the cutoff: long run low gray level emphasis, low gray level run emphasis, and the minimum. Short run low gray level emphasis was also often above the cutoff. While only features that passed the feature stability test were included in the analysis, we were interested in examining if these features’ poor performance in the IV_total_ and IV_residual_ tests could be attributed to other causes. Therefore, we re-examined the feature stability and found that these features were not as stable as many of the other features that also passed the test. There was no clear way to determine the cutoff for the feature stability test, but this indicates that the poor performance in the IV_total_ and IV_residual_ tests could be due to sensitivity of these features to the ROI placement.

Overall, there was very little to no improvement in the number of features above the cutoff when IV_residual_ was computed compared with IV_total_. There were fewer features above the cutoff for the controlled protocol compared with the local protocols except when thresholding, smoothing, and bit depth rescaling were used.

Twenty of the 24 pairwise t-tests of σ_β_, σ_γ_, and σ_ε_ between the controlled protocol and local chest protocol and between the controlled protocol and local head protocol were significant (p < 0.05). All comparisons between the controlled and local head protocol were not significant when thresholding and smoothing were applied as the preprocessing. Additionally, σ_ε_ was not significantly different between the controlled and local head protocol when thresholding, smoothing, and bit depth rescaling were applied as the preprocessing. Table 1 in the Supplemental Material shows the p-values for all comparisons.

Since there was a disproportionately high number of GE scanners, the linear mixed-effects models were also run with only the GE scanners. A pairwise t-test was run on σ_β_ and σ_ε_ between the models with all of the scanners and the models with only the GE scanners. There was a significant difference (p < 0.05) for 11 of the 24 comparisons between variabilities calculated from linear mixed-effects models with all scanners and models with GE scanners only. Table 2 in the Supplemental Material shows the p-values for all comparisons.

### Quality Assurance Using a Radiomics Phantom

The three cartridges with ABS had noticeable changes over the course of the study. The mean values of the cartridges over time are shown in Supplemental Figure 12. The three cartridges with ABS displayed a downward trend in mean value over time, while the other cartridges did not show any trend with time. Therefore, the three ABS cartridges were excluded from the QA analysis with a radiomics phantom.

The gray level run length matrix features had a disproportionately high number of scanners outside the established bounds; therefore, these features were not included in the QA analysis. Thus, 35 features with four preprocessing types were included in the QA test. Histograms of the number of scanners with the percentage of features outside the bounds set using the scaled patient standard deviation showed that many scanners had more than 20% of features outside the bounds, as shown in Supplemental Figure 13 for each of the rubber, dense cork, and hemp seed cartridges using the HNSCC and NSCLC patient cohorts.

**Figure 5:**
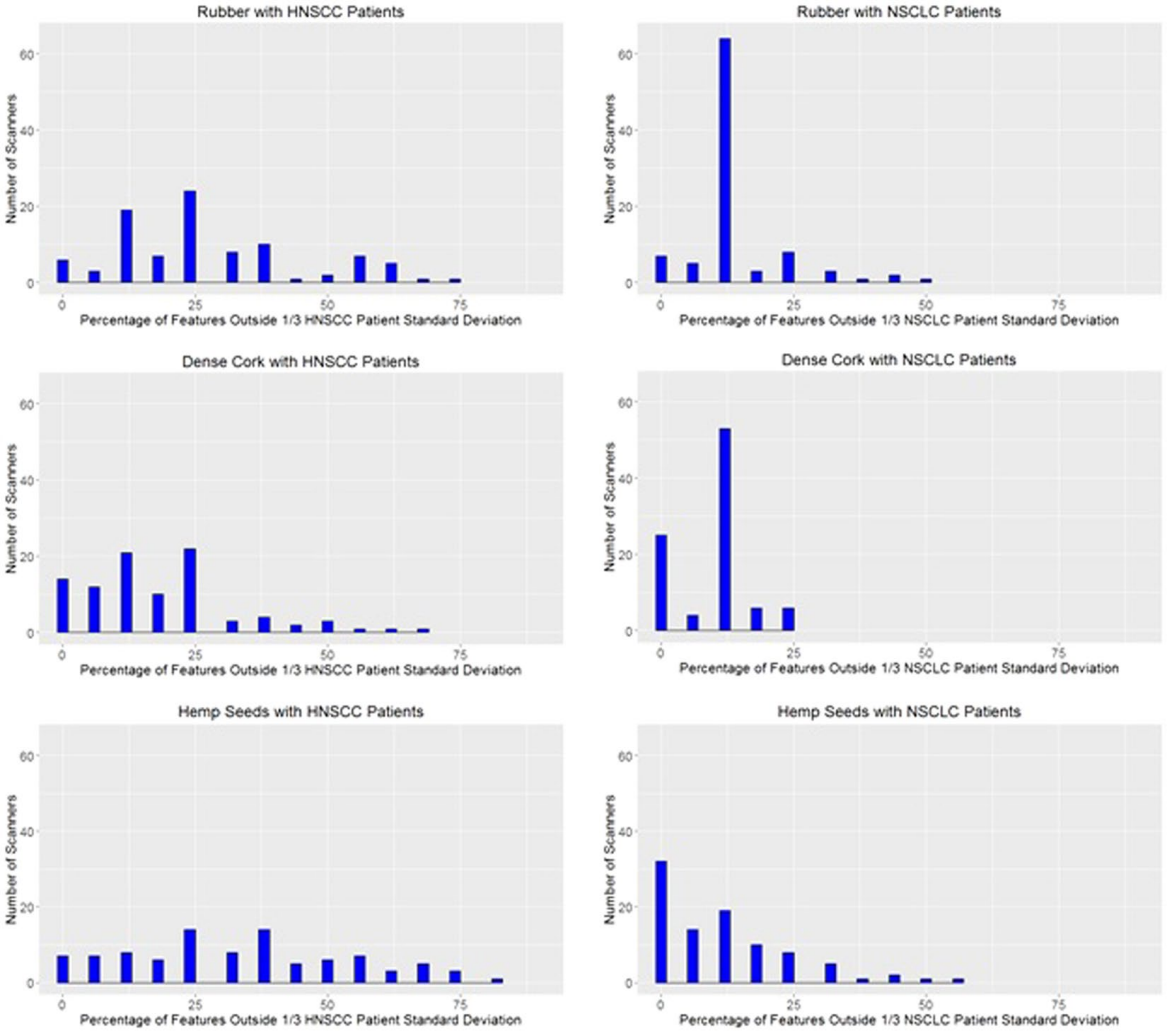
The percentages of features outside 1/3 of the scaled patient standard deviation for rubber, dense cork, and hemp seeds in the head and neck squamous cell carcinoma (HNSCC) patient cohort and the non-small cell lung cancer (NSCLC) patient cohort using the features correlated with patient survival in previous studies without non-robust features. More scanners had fewer features outside 1/3 of the patient standard deviation in the NSCLC patient cohort than the HNSCC patient cohort.

Not all features may be useful, as not all features have been correlated with patient outcomes. Therefore, a subset of features with associated preprocessing type were selected on the basis of studies by Fave *et al*. and Fried *et al*.^2,3^. The features and the preprocessing types that were correlated with patient survival on univariate analysis were included, which resulted in 26 features. Like the gray level run length matrix features, the features of auto correlation, correlation, sum average, sum variance, and the median had a disproportionately high number of scanners outside the bounds. Excluding the features that were shown to not be robust and excluding the gray level run length matrix features reduced the feature set to 16 features with their associated preprocessing types. These 16 features are listed in the Supplemental Table 3. Figure 5 shows histograms for percentages of features outside the bounds (similar to Supplemental Figure 13, but with the reduced set of features). More scanners had low percentage of features outside 1/3 of the scaled patient standard deviation in the NSCLC patient cohort than in the HNSCC patient cohort; this is discussed further in the Discussion section. One scanner consistently had the highest percentage of features outside the bounds. However, aside from this scanner, the scanners with the highest percentages of features outside the bounds were not consistent across the different materials.

## Discussion

This study showed that imaging variability exists but is not large compared with inter-patient variability for most features. A controlled scan can be helpful for reducing these uncertainties in prospective studies, as there was statistically significantly less variability in the controlled protocol scans than in the local protocol scans. The controlled protocol reduced the total variability by over 50% compared with both local chest and local head protocol scans. It is theoretically possible to correct for the manufacturer and the individual scanner. One possible way to do this is to use a phantom on each scanner to correct for all the factors that could impact the output of a scanner. If this were done perfectly, the imaging variability could be reduced by an additional 7–8% compared with the reduction due to implementing a controlled protocol.

The controlled protocol implemented in this study specified kernels for each manufacturer. Solomon *et al*. and Winslow *et al*. compared kernels on Siemens and GE^37,38^. Both found that the GE standard kernel was the closest match to the B31f or B31s kernel on Siemens, which agrees with our controlled protocol. Additionally, Shafiq-ul-Hassan *et al*. recently demonstrated the feasibility of correcting for the different kernels, achieving improvements in feature robustness by 30–78%^39^. Our goal in this study was to harmonize the kernels across manufacturers such that the kernel did not affect the imaging variability. However, including this new correction technique may reduce imaging variability further.

Gray level run length matrix features had high feature variability when ROIs were moved. Additionally, these features had the highest imaging variability. We believe that these results are due to the current construction of these features. Examining low gray level run emphasis demonstrates this issue. Low gray level run emphasis is defined as

8 $$LGRE=\frac{1}{{n}_{r}}{\sum }_{i=1}^{M}\frac{{p}_{g}(i)}{{i}^{2}}$$

where n_r_ is the total number of runs, M is the total number of gray levels, i is the gray level, and

9 $${p}_{g}(i)={\sum }_{j=1}^{N}p(i,j)$$

is the sum distribution of the number of runs with gray level i, run length j, maximum run length N, and run-length matrix p(i, j). A slight shift in the distribution of gray levels within the ROI can significantly impact the feature value as the range of the summations remain the same but p(i, j) changes, thus impacting the feature value. Thus we recommend that these features not be used until these issues can be resolved. This problem may be why gray level run length matrix features have not come out in the final models in many studies.

Many of the features showed a correlation between feature value and image thickness that must be considered. Also, the slope of the fixed-effects term for the image thickness was generally the same for a given feature across all models, even in the controlled protocol scans where there were only two image thickness values, indicating the strength of this relationship. This agrees with several studies that have demonstrated the relationship between radiomics features and image thickness^16–18,40^. However, the high ICC values indicate that the feature value correlation with image thickness cannot be fixed by resampling the image and thus cannot be fixed for retrospective scans for this particular phantom study. When the range of resampled image thickness values was decreased (i.e. not including thicknesses above 5 mm), the ICC values remained high. Noise characteristics were not included in this part of the study which can affect feature values as thicker slices can introduce less noise than thinner slices. Even given the limitations of this study, these results indicate that this effect cannot be compensated for after reconstruction with resampling for this phantom study. This is in contrast to the studies by Shafiq-ul-Hassan *et al*. and Larue *et al*. who found that resampling to an arbitrarily chosen standard voxel size improved feature reproducibility^16,20^. Therefore, in this study there is a need to control the image thickness as resampling to a variety of image thickness values did not change the feature value, and thus, we recommend controlling image thickness in prospective studies to eliminate this feature value dependence. If the image thickness cannot be completely controlled, the range of image thicknesses used within a study cohort should be limited to reduce this effect.

The importance of a controlled protocol for prospective studies was also demonstrated through the linear mixed-effects models. There was significantly less variability in the controlled protocol scans compared with the local protocol scans. Furthermore, the total variability (Table 2) does not include the contribution from the fixed-effect term for image thickness, which would increase imaging variability. Reducing the uncertainty is a crucial step in moving forward with radiomics studies, as reduced uncertainty allows more levels of stratification in prognostic models and enables the movement towards individual prognostic models instead of sorting patients into groups. The manufacturer-wise variation was reduced when a controlled scan was implemented because imaging parameters were harmonized. Many local protocols use the standard kernel, but this kernel is not the best match across different manufacturers. The controlled scan also demonstrated more benefit than post-processing correction for the manufacturer and individual scanner. Radiomics has traditionally been conducted on standard of care imaging, but the large improvements of a controlled protocol demonstrated in this study show the potential importance of such a controlled scan. Thus, efforts should be made to implement a controlled protocol for prospective radiomics studies, and only patients whose imaging parameters match the controlled protocol should be selected in retrospective studies. Studies by Mackin *et al*.^24^ and Fave *et al*.^33^ have shown that tube current and tube voltage do not significantly impact the majority of radiomics features. Therefore, the reconstruction settings dominate the imaging variability and most of the benefit of the controlled scan can be achieved using an additional radiomics reconstruction resulting in no extra dose to the patient.

This study uses the second version of the radiomics phantom. The lessons learned from the first phantom, used in several studies^16,22^, led to this new, improved phantom. The buildup was one considerable difference between the phantoms. Buildup was added to make the phantom more realistic. Also, only the rubber and cork cartridges were kept from the first phantom, as features measured from these cartridges more closely matched NSCLC patient features than did features from other cartridges in the first phantom. In this phantom, we added hemp seed and ABS cartridges, and we have learned that for future phantoms, ABS cartridges should not be used, as they change over time. The cartridges that were added matched features calculated from patients better and produced a more realistic range of textures. While three of the cartridges changed over time and thus are not optimal options for future work, removing these from the linear mixed-effects models did not change conclusions.

Almost all of the scanners in this study followed established QA protocols. However, in spite of this there were still large imaging variabilities. Therefore, there may be a need for radiomics QA and we demonstrated the potential for a radiomics QA process. The different materials identified different scanners with large percentage of features outside the established bounds, which indicates that a radiomics QA phantom may not be feasible with only one material. The choice of 1/3 in establishing the bounds was arbitrary. The cutoff for the percentage of features failed that would be acceptable to pass the QA process depends on the bounds chosen. When the features found to be correlated with patient survival by Fave *et al*. and Fried *et al*.^2,3^ were used, the histograms of the number of scanners with features outside the bounds decreased, likely because those features are more robust. While studies have found that a radiomics signature developed from NSCLC patients can be used to predict survival in head and neck cancer patients^11,41^, there are distinct feature clusters for the lung and the head and neck cancer patient cohorts^5^. Our patient sets also showed different feature distributions for lung and head and neck patient cohorts, which contributed to the difference in QA results. Therefore, for QA purposes, a distinct radiomics signature should be selected for each cancer site to be credentialed.

There are several limitations to this study. First, the phantom was not imaged by a single user; therefore, there may be some added variability due to different users. Secondly, the phantom materials are not the same as human tissue. Dense cork and rubber have been previously shown to have radiomics feature spectrums similar to those of NSCLC patients^22^, and these cartridges have effective atomic numbers close to those of human tissues^42–44^. Using patients for these studies is not feasible; therefore, these materials are a close match to human tissues, and results derived from them can be applied to patient CT scans. Additionally, the same phantom was used for chest and head scans. The dimensions of the phantom were designed for chest imaging. Visual inspection of the images did not yield any artifacts specific to the head protocols. While not optimized for head imaging, this phantom still provides valuable information on the radiomics feature variability of these protocols.

Also, there was not an even distribution of scanners by manufacturer. There was a disproportionately high number of GE CT scanners, and it is unknown whether our sample of scanners accurately represents the distribution of scanners in clinical use, as these data are not available. When GE scanners alone were run through the linear mixed-effects model, some variabilities were statistically significantly different between the GE scanners alone and between all scanners. This difference may point to there being scanner-wise variability differences between manufacturers which was not accounted. This was due to the limited number of scanners outside GE which is a limitation of this study. The sample of scanners selected were acquired in Dallas, San Antonio, Houston, Galveston, Baton Rouge, and New Orleans thru proximity and personal contacts. As this sample only constitutes scanners from Texas and Louisiana, the manufacturer distribution may look different in other parts of the USA or in other countries. Additionally, the patient scans used were from selected scanners using well-specified imaging parameters. This may not represent the true inter-patient variation that may exist in a large radiomics study. However, as these patient scans were well controlled, this provides a conservative estimate of the imaging variability effect within patient cohorts. The results from IV_total_ and IV_residual_ are promising given that this may be a conservative estimate and within a larger patient cohort even fewer features may be adversely affected due to larger inter-patient variation.

## Conclusion

A controlled protocol substantially reduces imaging variability compared with local protocols, as the controlled protocol can reduce the total variability by more than 50%. Thus, controlled protocols should be used for radiomics studies. Most of this benefit can be achieved by an extra radiomics reconstruction resulting in no additional dose to the patient. Correcting for the manufacturer and individual scanner can also yield an additional benefit.

## Electronic supplementary material

Supplemental Material

Supplementary Dataset 1

Supplementary Dataset 2

## Data Availability

The datasets generated during and/or analyzed during the current study are available from the corresponding author on reasonable request.
