# Supplementary material for: Comprehensive Investigation on Controlling for CT Imaging Variabilities in Radiomics Studies
Source: Sci Rep. 2018 Aug 29;8:13047. doi: 10.1038/s41598-018-31509-z (PMC6115360; doi:10.1038/s41598-018-31509-z)
Supplement: Supplementary file 1 — Supplemental Material [file 41598_2018_31509_MOESM1_ESM.pdf]

SUPPLEMENTARY MATERIAL

Comprehensive Investigation on Controlling for Inter-scanner Variabilities in Radiomics Studies

Rachel B. Ger[,1,2*], Shouhao Zhou[2,3], Pai-Chun Melinda Chi[1], Hannah J. Lee[1], Rick R. Layman[2,4], A. Kyle Jones[2,4], David L. Goff[5], Clifton D. Fuller[2,6], Rebecca M. Howell[1,2], Heng Li[1,2], R. Jason Stafford[2,4], Laurence E. Court[1,2,4], Dennis S. Mackin[1,2]

[1]Department of Radiation Physics, The University of Texas MD Anderson Cancer Center, Houston, Texas

[2]MD Anderson Cancer Center UTHealth Science Center at Houston Graduate School of Biomedical Sciences, Houston, Texas

[3]Department of Biostatistics, The University of Texas MD Anderson Cancer Center, Houston, Texas

[4]Department of Imaging Physics, The University of Texas MD Anderson Cancer Center, Houston, Texas

[5]Medical & Radiation Physics, Inc., San Antonio, Texas

[6]Division of Radiation Oncology, The University of Texas MD Anderson Cancer Center, Houston, Texas

*Corresponding author: rbger@mdanderson.org

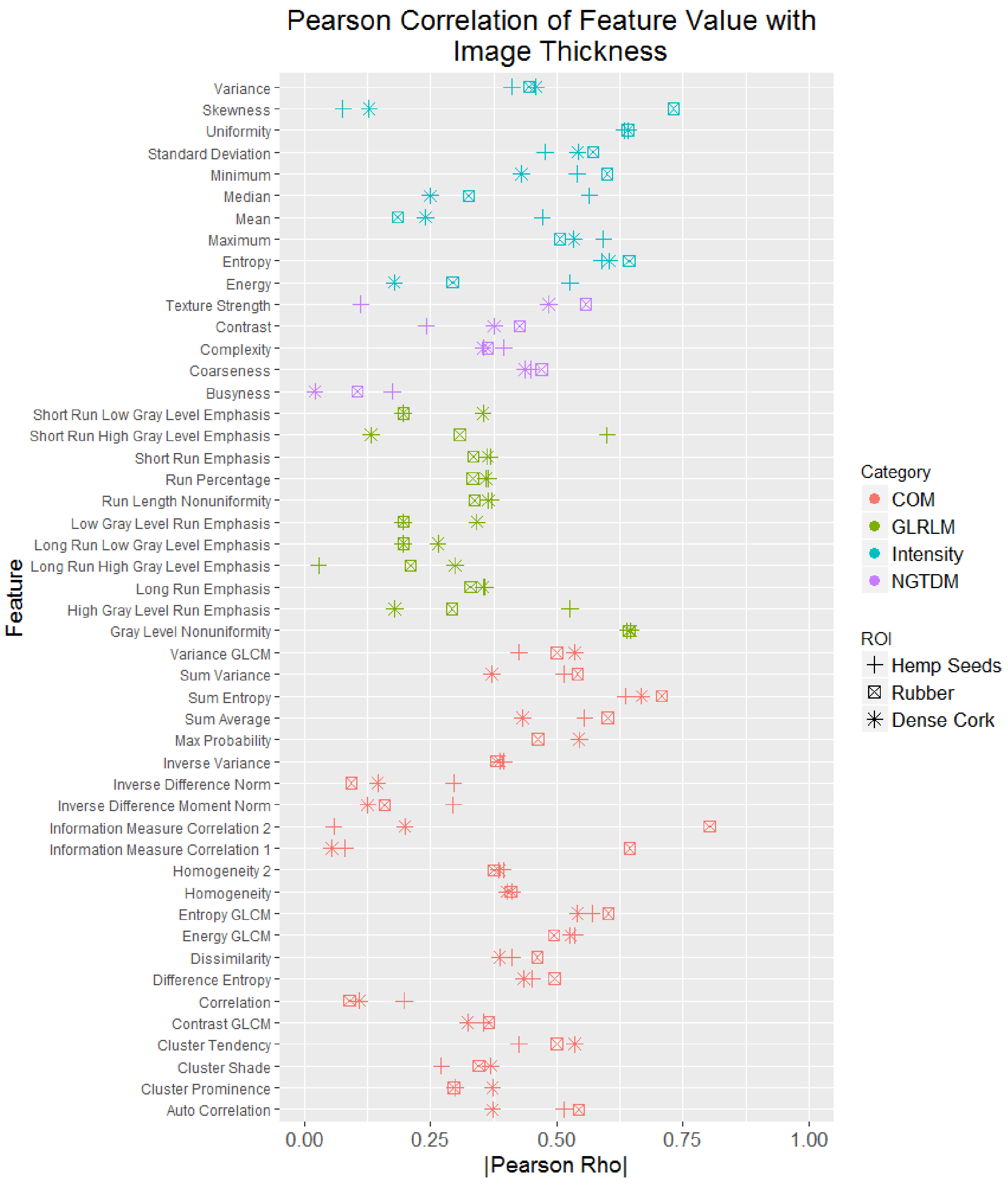

Supplemental Figure 1. Absolute value of the Pearson correlation rho for the correlation between feature value and image thickness for each region of interest (ROI). Each ROI is a different shape. Each category of feature is a different color. The correlation varies between and within features depending on

the ROI. COM: gray level co-occurrence matrix, GLCM: gray level co-occurrence (used when there are features with the same name in different categories to differentiate them), GLRLM: gray level run length matrix, NGTDM: neighborhood gray tone difference matrix.

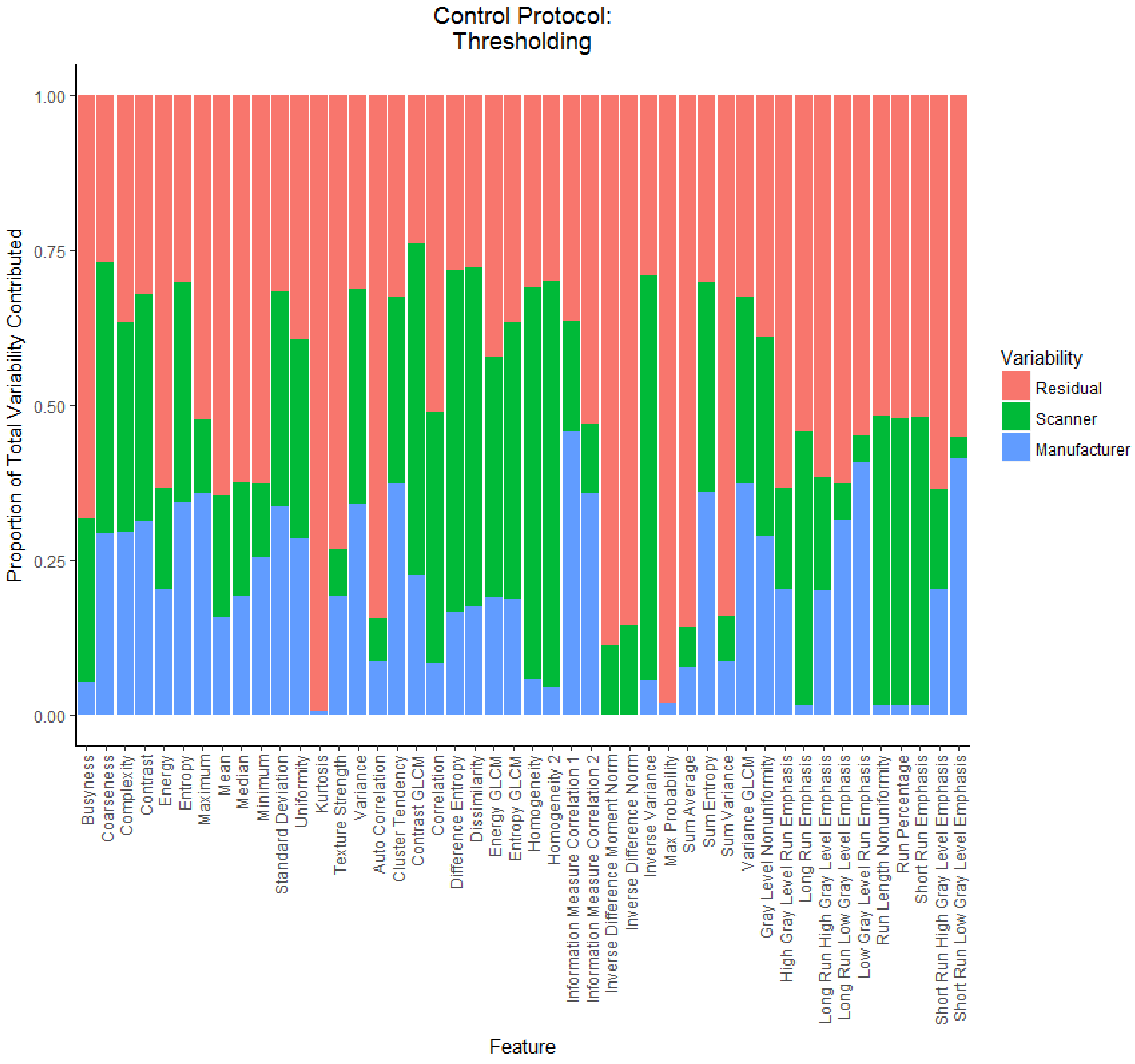

Supplemental Figure 2. Bar plots of the relative contribution of the scanner wise variability, manufacturer wise variability, and residual variability for each feature using thresholding calculated on the control protocol.

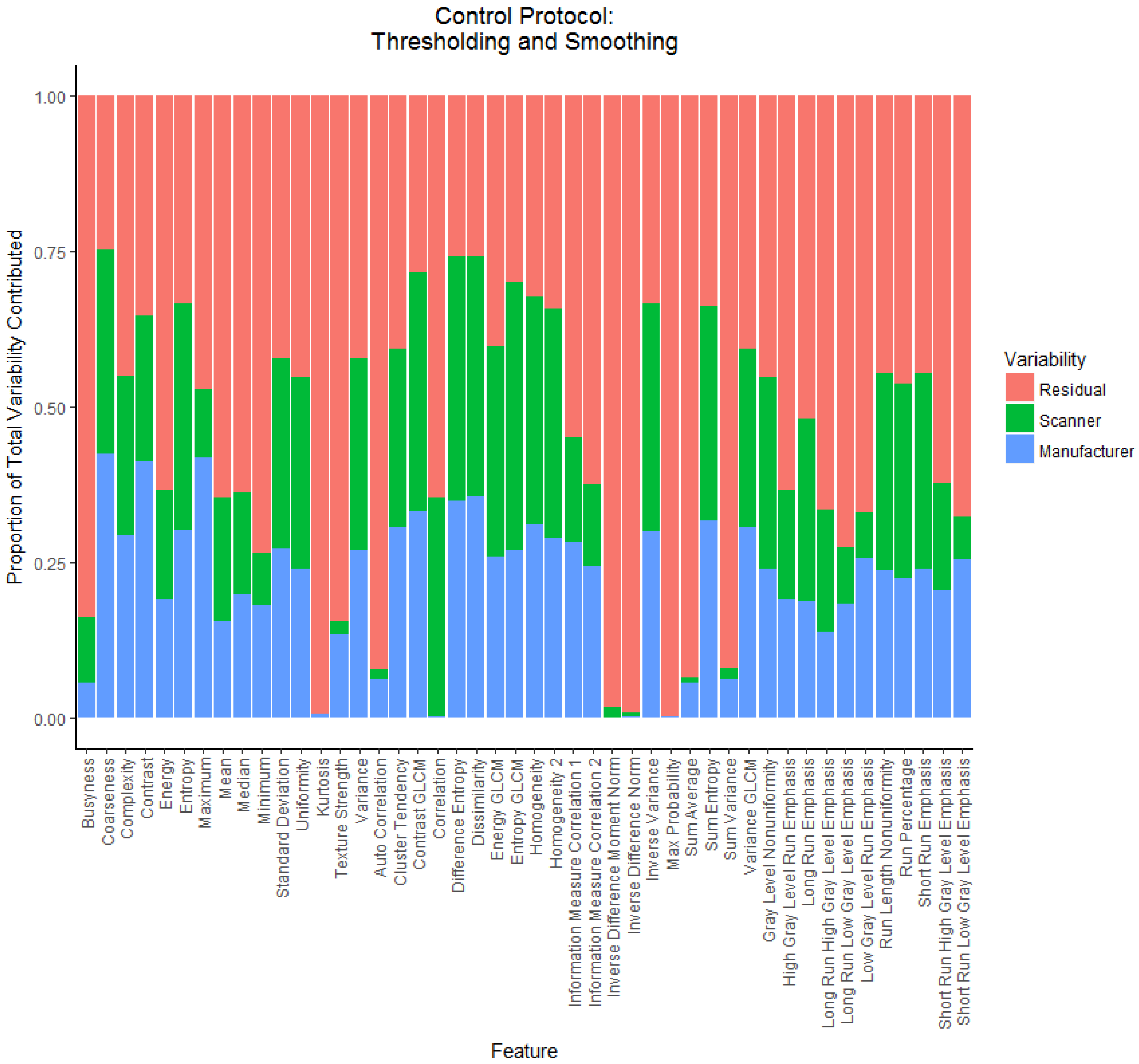

Supplemental Figure 3. Bar plots of the relative contribution of the scanner wise variability, manufacturer wise variability, and residual variability for each feature using thresholding and smoothing calculated on the control protocol.

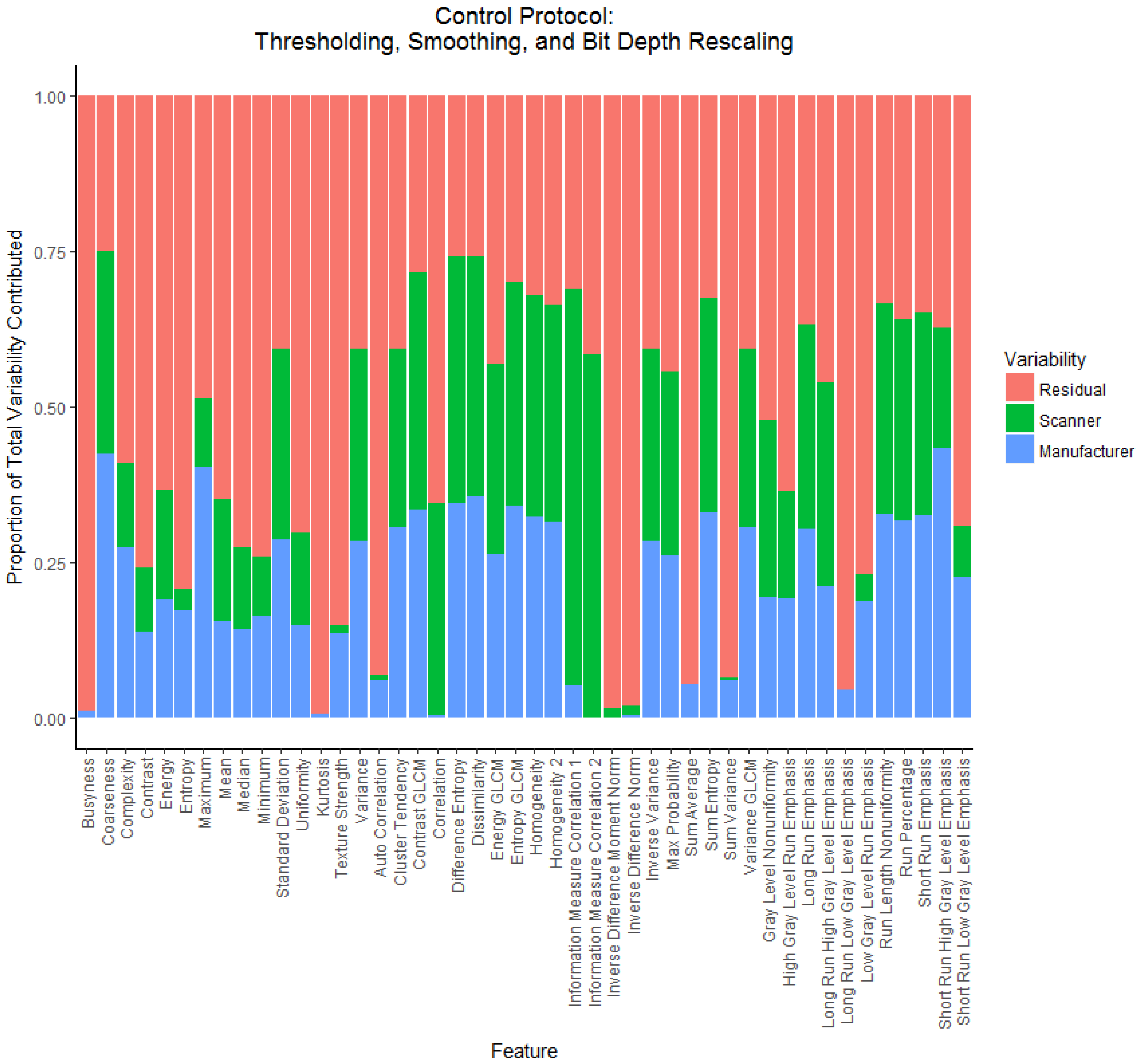

Supplemental Figure 4. Bar plots of the relative contribution of the scanner wise variability, manufacturer wise variability, and residual variability for each feature using thresholding, smoothing, and bit depth rescaling calculated on the control protocol.

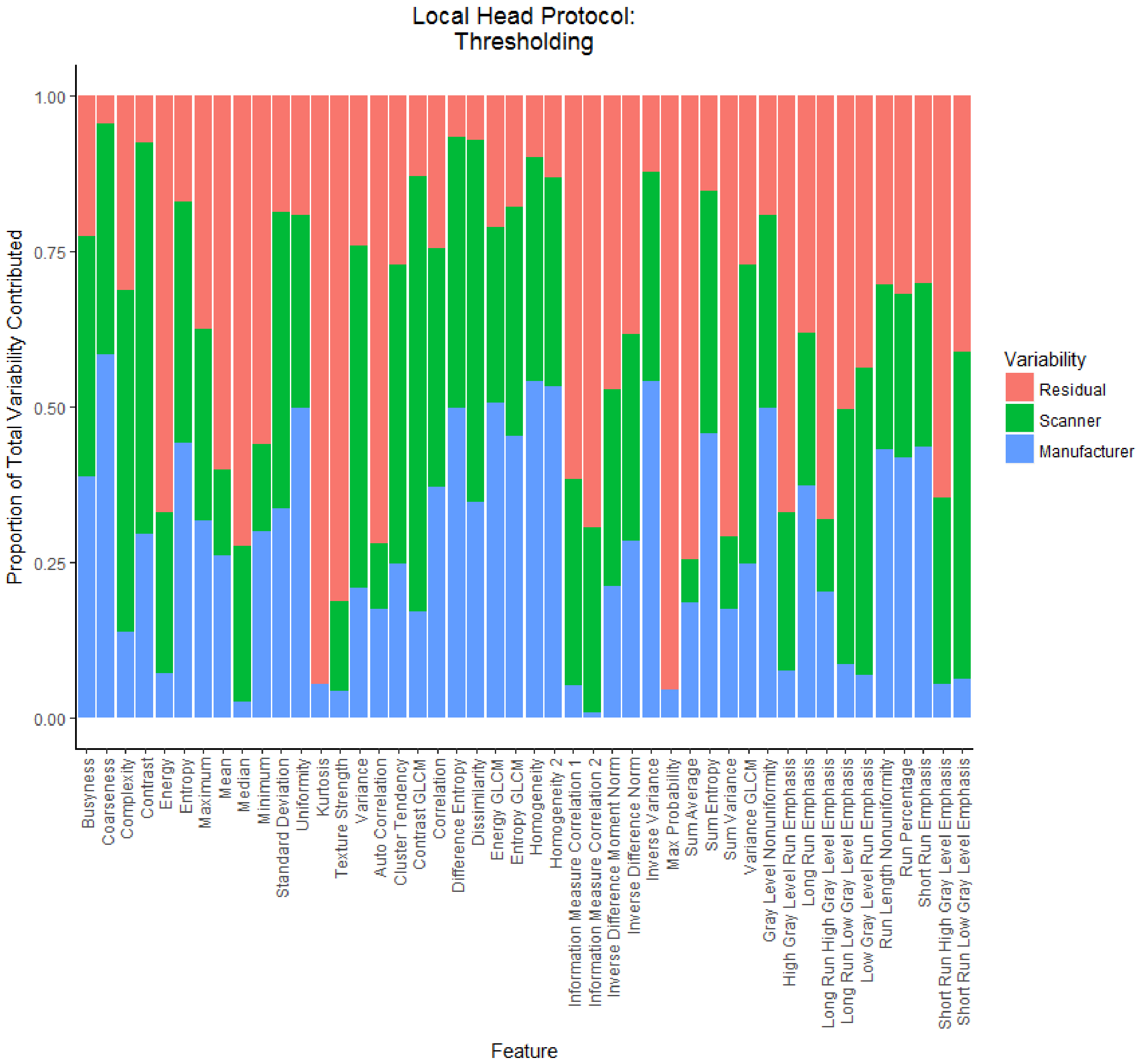

Supplemental Figure 5. Bar plots of the relative contribution of the scanner wise variability, manufacturer wise variability, and residual variability for each feature using thresholding calculated on the local head protocol.

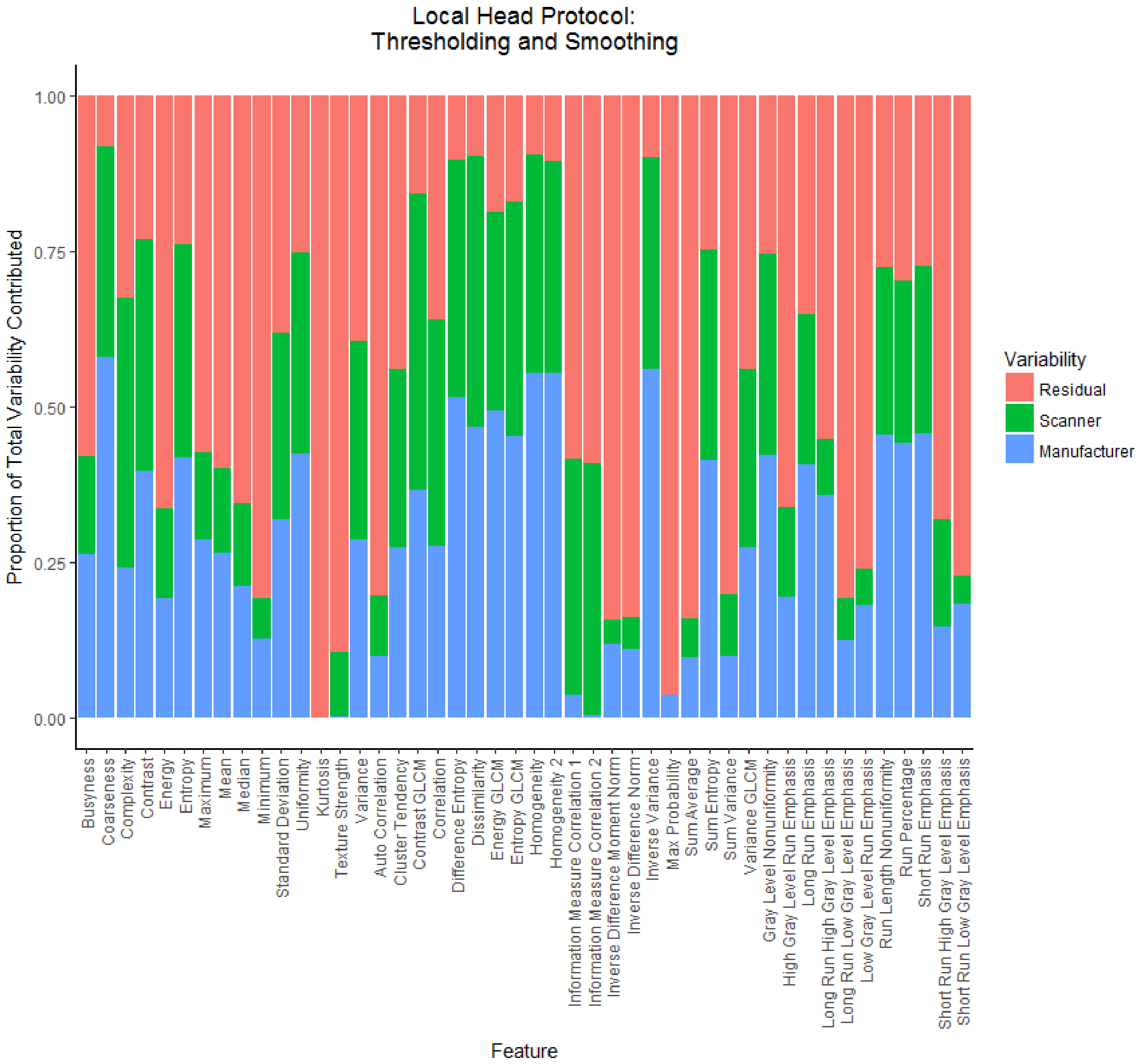

Supplemental Figure 6. Bar plots of the relative contribution of the scanner wise variability, manufacturer wise variability, and residual variability for each feature using thresholding and smoothing calculated on the local head protocol.

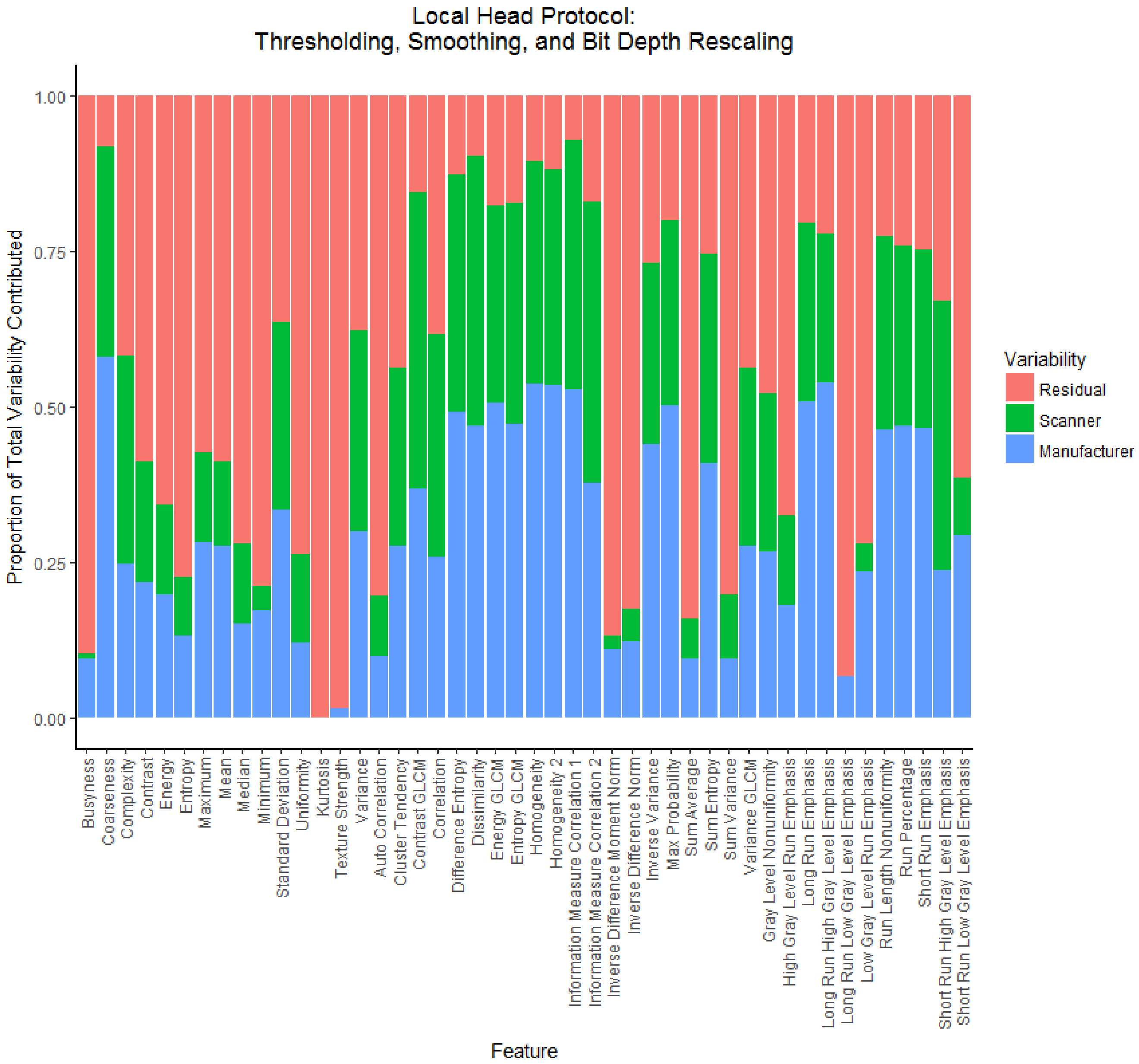

Supplemental Figure 7. Bar plots of the relative contribution of the scanner wise variability, manufacturer wise variability, and residual variability for each feature using thresholding, smoothing, and bit depth rescaling calculated on the local head protocol.

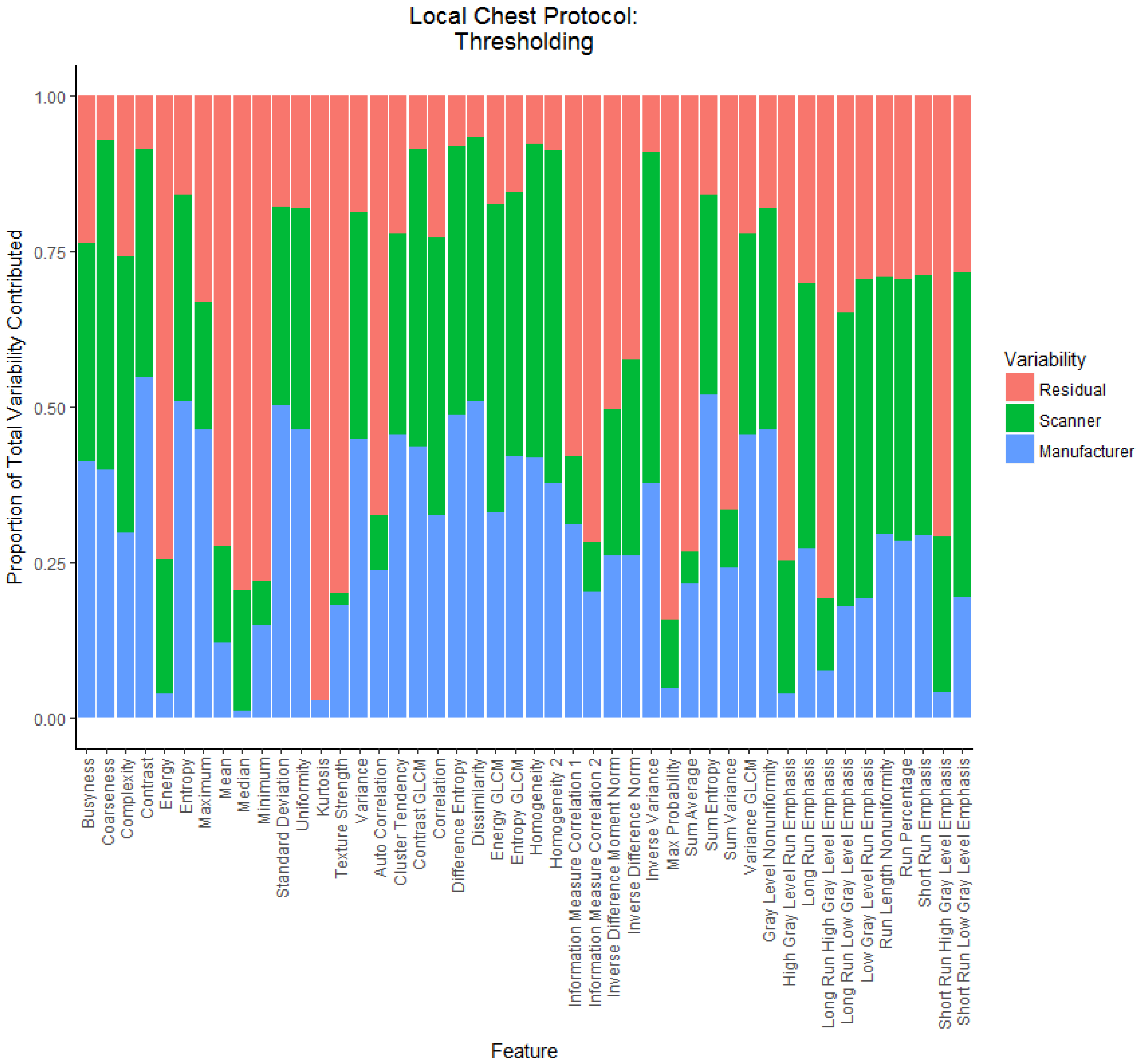

Supplemental Figure 8. Bar plots of the relative contribution of the scanner wise variability, manufacturer wise variability, and residual variability for each feature using thresholding calculated on the local chest protocol.

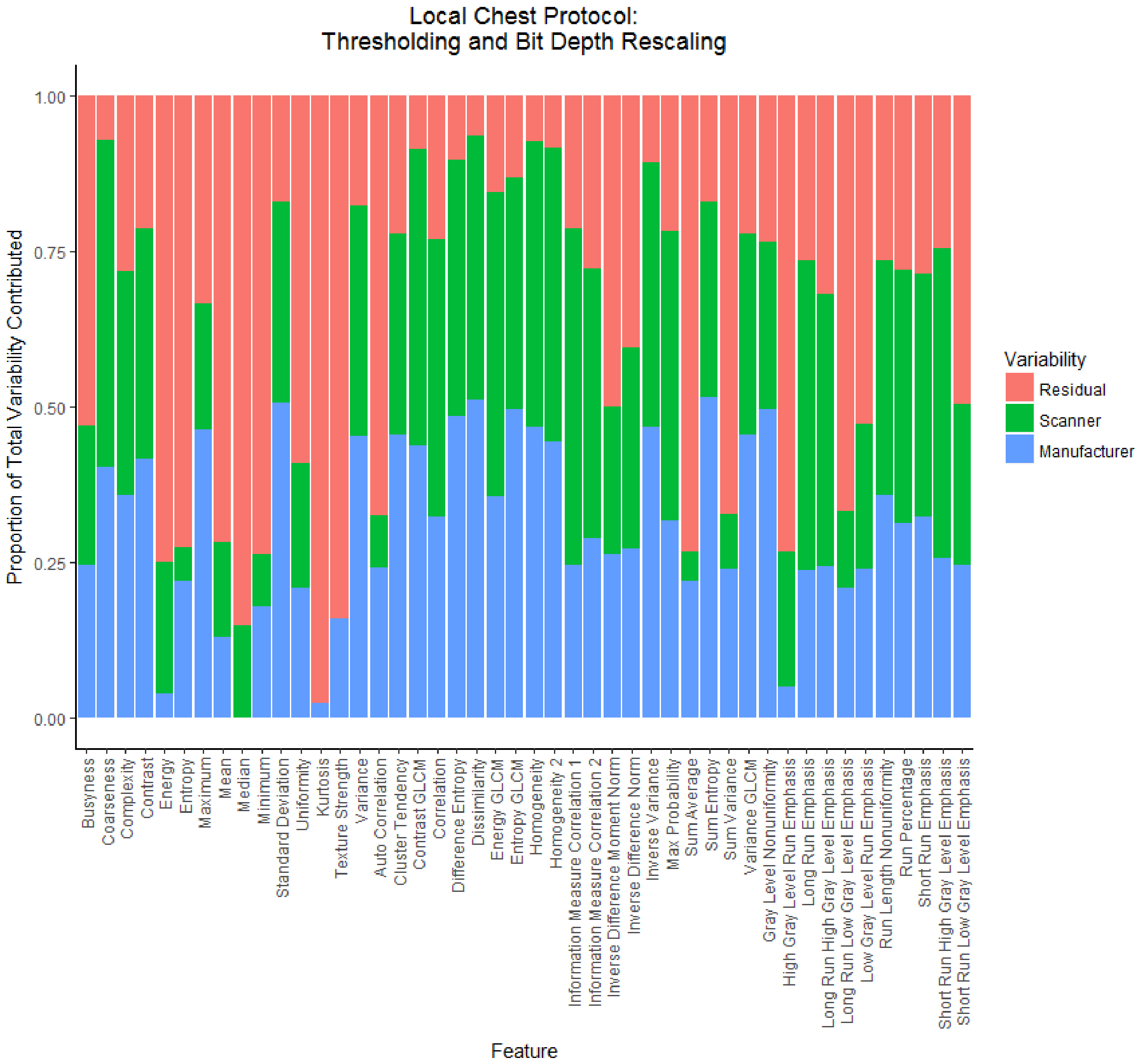

Supplemental Figure 9. Bar plots of the relative contribution of the scanner wise variability, manufacturer wise variability, and residual variability for each feature using thresholding and bit depth rescaling calculated on the local chest protocol.

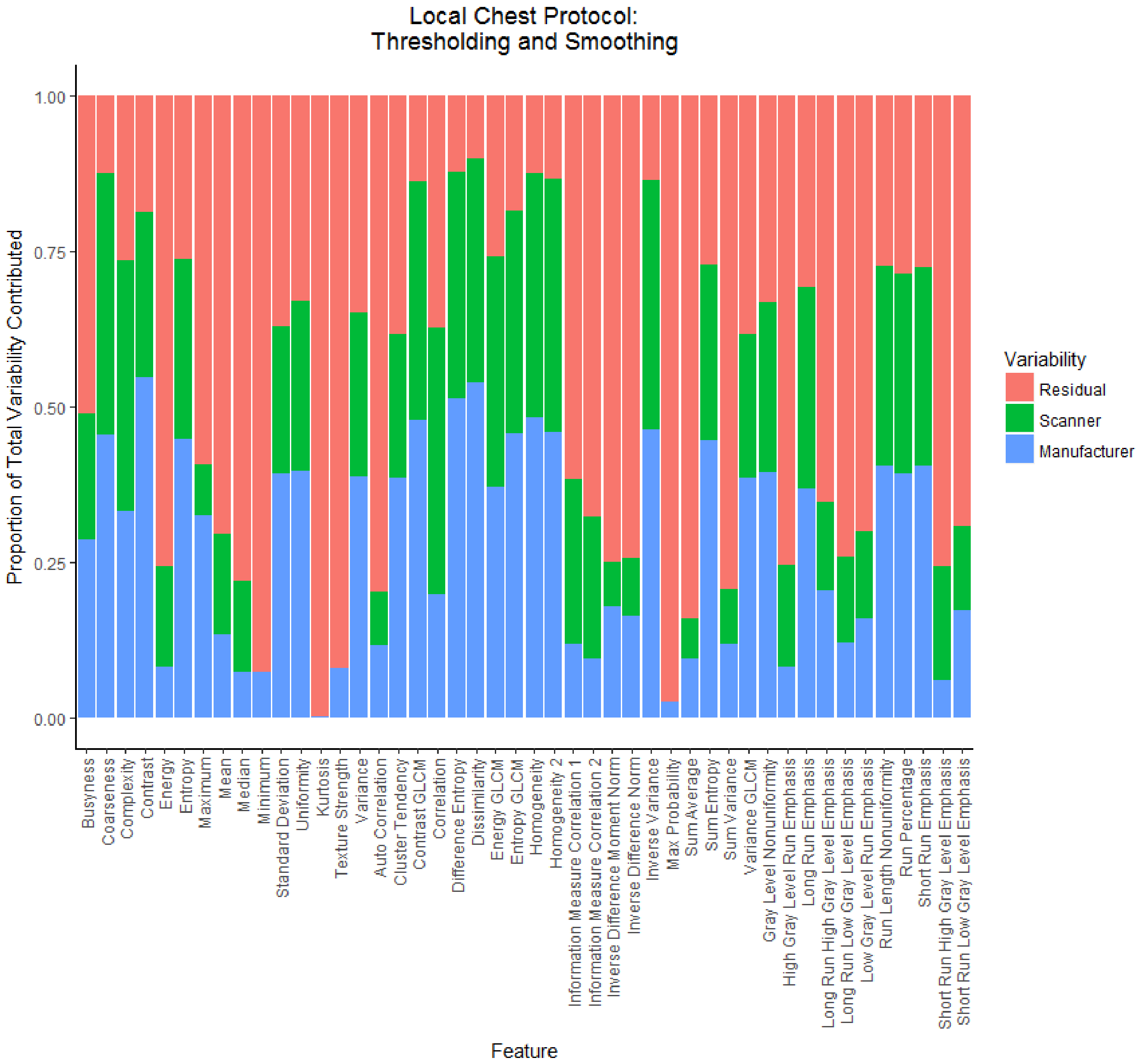

Supplemental Figure 10. Bar plots of the relative contribution of the scanner wise variability, manufacturer wise variability, and residual variability for each feature using thresholding and smoothing calculated on the local chest protocol.

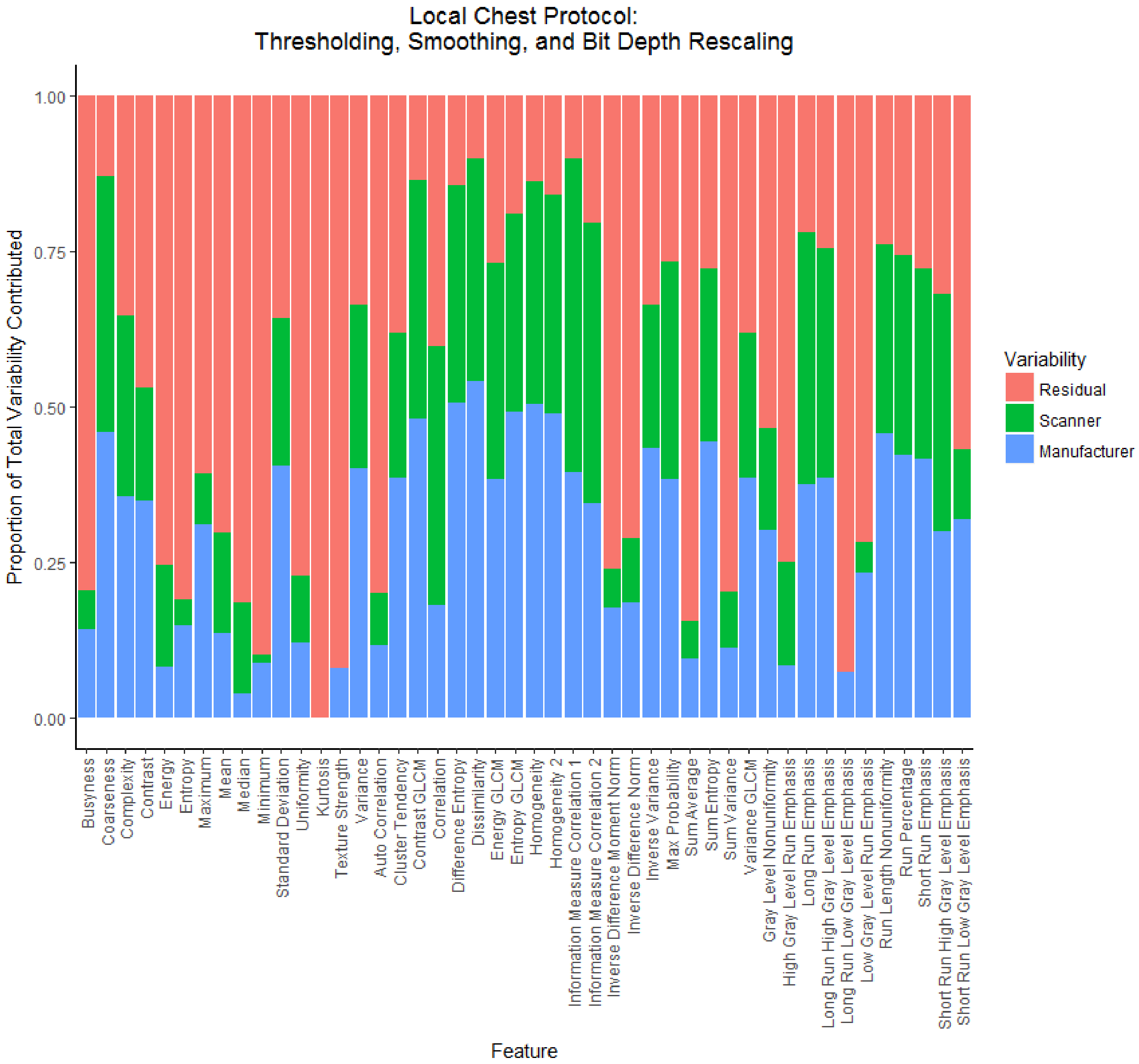

Supplemental Figure 11. Bar plots of the relative contribution of the scanner wise variability, manufacturer wise variability, and residual variability for each feature using thresholding, smoothing, and bit depth rescaling calculated on the local chest protocol.

Table 1. P-values for One Sided Pairwise T-tests between Control and Local Protocol Scans

	Control vs Chest Protocol				Control vs Head Protocol			
	Thresholding	Thresholding and Smoothing	Thresholding and Bit Depth Rescaling	Thresholding, Smoothing, and Bit Depth Rescaling	Thresholding	Thresholding and Smoothing	Thresholding and Bit Depth Rescaling	Thresholding, Smoothing, and Bit Depth Rescaling
σ_β	0.037	0.032	0.00073	0.0042	0.035	0.54	0.0015	0.0024
σ_γ	0.027	0.018	0.00022	0.00049	0.0072	0.90	0.0000093	0.000097
σ_ε	0.038	0.046	0.0050	0.0049	0.036	0.42	0.0014	0.073

σ_β: scanner-wise variability, σ_γ: manufacturer-wise variability, σ_ε: residual variability

Table 2. P-values for Pairwise T-tests between Variability from Model with All Manufacturers vs GE Only

	Scanner-wise Variability			Residual variability		
	Control Protocol	Local Chest Protocol	Local Head Protocol	Control Protocol	Local Chest Protocol	Local Head Protocol
Thresholding	0.088	0.084	0.076	0.29	0.070	0.071
Thresholding and Smoothing	0.45	0.0078	0.0024	0.051	0.13	0.48
Thresholding and Bit Depth Resampling	0.93	0.21	0.025	0.0022	0.028	0.0081
Thresholding, Smoothing, and Bit Depth Resampling	0.049	0.00022	0.0016	0.0024	0.083	0.0014

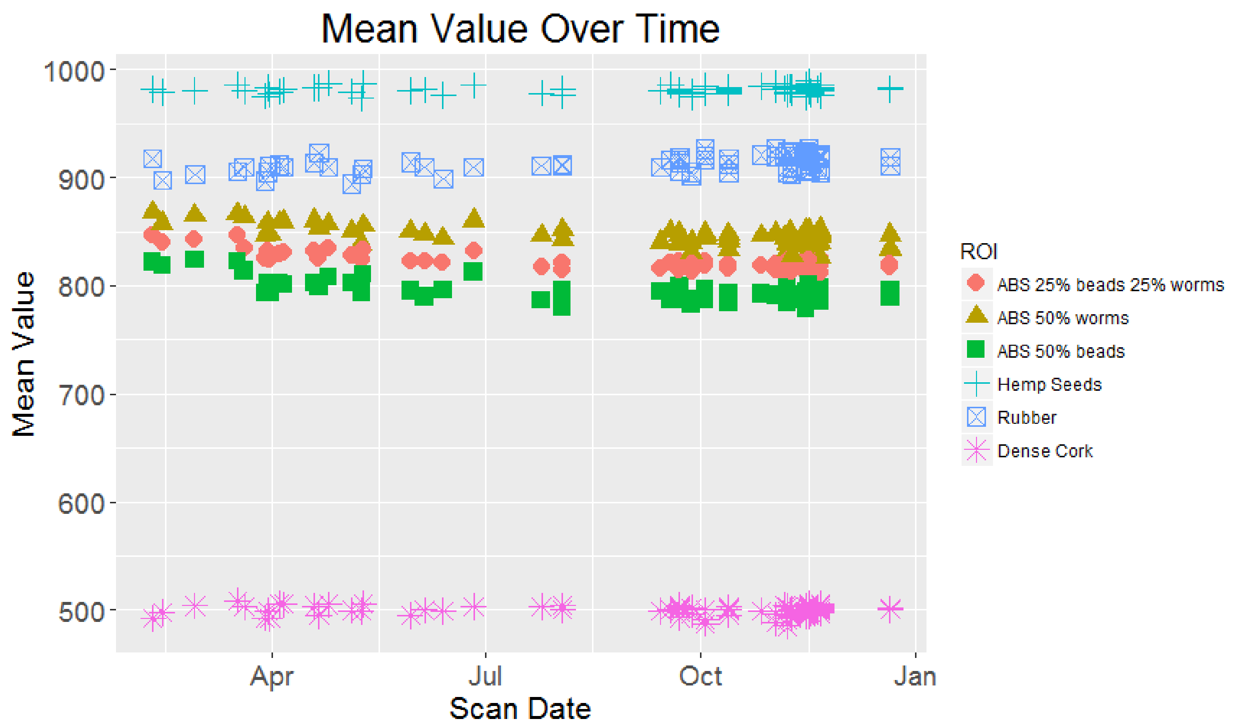

Supplemental Figure 12. Mean value over time for each ROI, shown as a different colored and shaped point. The three cartridges with ABS demonstrated a downward trend with time while the other cartridges did not demonstrate any trend with time.

Percentage of Features Outside 1/3 Scaled Patient Standard Deviation Using All Robust Features

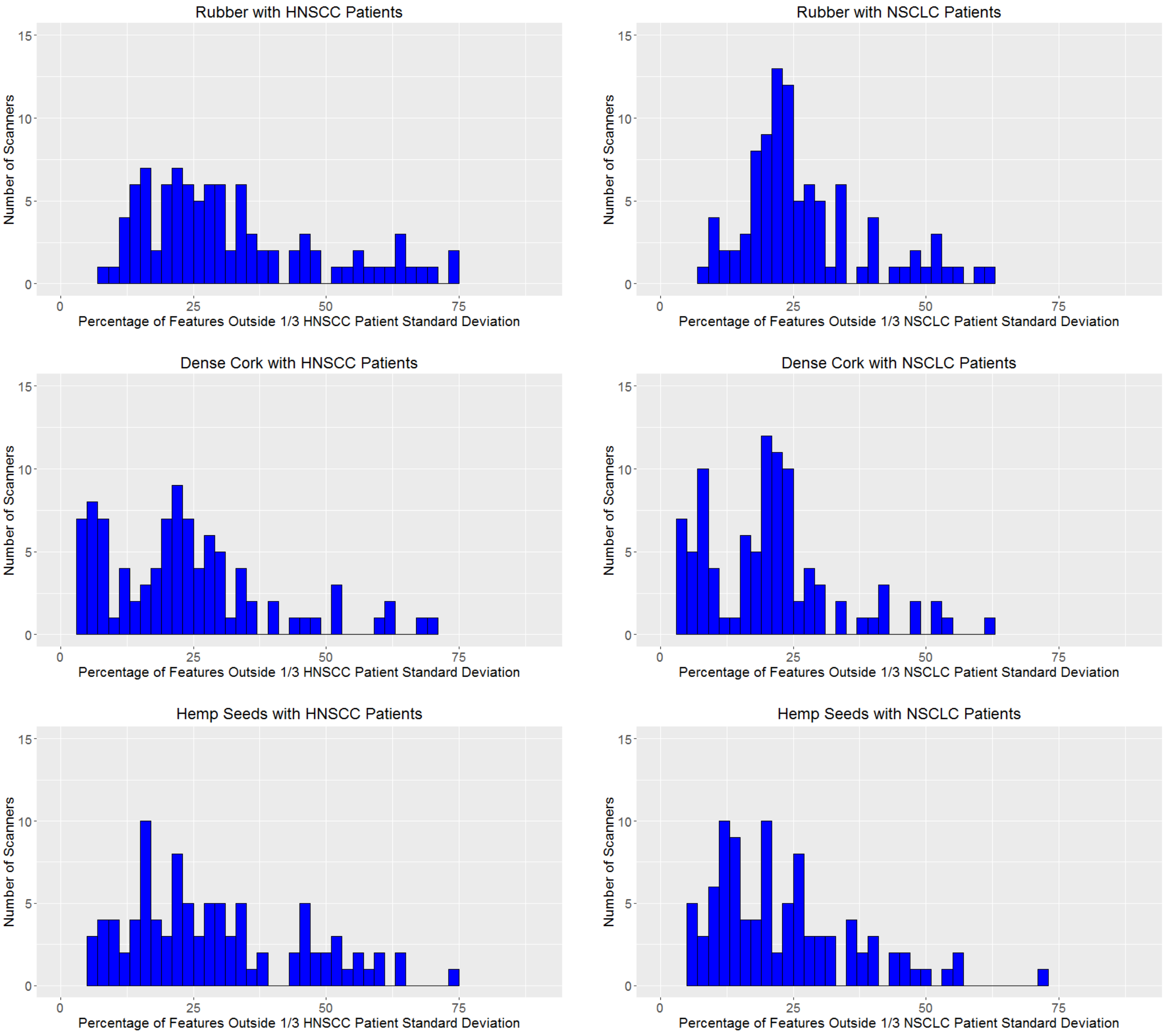

Supplemental Figure 13. The percentage of features outside 1/3 scaled patient standard deviation for rubber, dense cork, and hemp seeds using the head and neck squamous cell carcinoma (HNSCC) patient cohort and the non-small cell lung cancer (NSCLC) patient cohort. For all plots, there are a large percentage of features outside of a third of the scaled patient standard deviation.

Table 3. Preprocessing and Features From Previous Studies Used for QA Analysis

Preprocessing	Feature Group	Feature
Thresholding and Smoothing	NGTDM	Texture Strength
Thresholding and Smoothing	IH	Variance
Thresholding and Smoothing	IH	Standard Deviation
Thresholding and Smoothing	IH	Mean
Thresholding and Smoothing	IH	Kurtosis
Thresholding, Smoothing, and Bit Depth Resampling	IH	Entropy
Thresholding and Smoothing	IH	Energy
Thresholding and Smoothing	GLCM	Inverse Difference Norm
Thresholding and Smoothing	GLCM	Inverse Difference Moment Norm
Thresholding	GLCM	Information Measure Correlation 1
Thresholding	GLCM	Information Measure Correlation 2
Thresholding and Smoothing	GLCM	Dissimilarity
Thresholding, Smoothing, and Bit Depth Resampling	GLCM	Difference Entropy
Thresholding and Smoothing	GLCM	Contrast
Thresholding	NGTDM	Busyness
Thresholding	IH	Kurtosis

NGTDM: neighborhood gray tone difference matrix; IH: intensity histogram; GLCM: gray level co-occurrence matrix
